# Supplementary material for: A Paradoxical Role for Regulatory T Cells in the Tumor Microenvironment of Pancreatic Cancer
Source: Cancers (Basel). 2022 Aug 10;14(16):3862. doi: 10.3390/cancers14163862 (PMC9405872; doi:10.3390/cancers14163862)
Supplement: Supplementary file 1 [file cancers-14-03862-s001.zip › cancers-1824358-supplementary.pdf]

Table S1. Overview of differentially expressed genes (DEGs), within the distinct clusters found, of the respective subtypes after unsupervised analysis of gene expression profiles.
EMT cluster

Ensmbl_ID	Gene	logFC	AveExpr	t	P.Value	adj.P.Val	B
ENSG00000083782	EPYC	4,075679	1,754344	6,408765	1,47E-08	2,21E-05	9,277168
ENSG00000186081	KRT5	4,000901	1,4913515	5,517208	5,45E-07	0,000161	5,944353
ENSG00000230937	MIR205HG	3,971296	-1,067328	4,943977	5,05E-06	0,0005689	3,583555
ENSG00000205420	KRT6A	3,942058	2,9927075	4,73515	1,11E-05	0,0009042	3,191866
ENSG00000167656	LY6D	3,63424	0,8489659	5,20205	1,88E-06	0,0003218	4,766411
ENSG00000166535	A2ML1	3,222218	1,0198856	4,172409	8,53E-05	0,0032694	1,356381
ENSG00000186847	KRT14	3,122534	0,2845967	4,936424	5,20E-06	0,0005732	3,794841
ENSG00000105141	CASP14	2,954604	-1,902628	3,727927	0,000388	0,0086393	-0,12115
ENSG00000181143	MUC16	2,933653	4,5612173	3,948541	0,000185	0,0053814	0,418623
ENSG00000275216	NA	2,811584	-0,370639	4,684329	1,34E-05	0,0010274	2,883297
ENSG00000171401	KRT13	2,581586	1,2060024	4,01712	0,000146	0,0046423	0,873353
ENSG00000267013	LINC01929	2,554296	-0,333194	5,488397	6,11E-07	0,0001747	5,54947
ENSG00000172061	LRRC15	2,516502	4,0110585	5,351782	1,05E-06	0,0002356	5,363201
ENSG00000255398	HCAR3	2,442323	-0,592241	5,17206	2,11E-06	0,0003514	4,42887
ENSG00000142619	PADI3	2,431548	-0,081473	3,958163	0,000179	0,0052913	0,674273
ENSG00000182782	HCAR2	2,250459	1,1103728	5,129437	2,48E-06	0,0003804	4,537959
ENSG00000169469	SPRR1B	2,236227	0,7885547	3,513909	0,000779	0,0138108	-0,59974
ENSG00000166342	NETO1	2,223819	-1,030851	4,510233	2,54E-05	0,0015587	2,237886
ENSG00000142623	PADI1	2,214935	5,188886	3,64874	0,000504	0,0103977	-0,57259
ENSG00000087128	TMPRSS11E	2,197056	-0,826753	3,601241	0,000588	0,0115026	-0,37114
ENSG00000137745	MMP13	2,175325	1,3446402	4,628621	1,65E-05	0,0011856	2,840251
ENSG00000205426	KRT81	2,124414	-0,271638	4,323016	5,00E-05	0,0023346	1,761188
ENSG00000138271	GPR87	2,071233	2,2110859	3,73592	0,000378	0,0084662	-0,017
ENSG00000088726	TMEM40	2,046272	0,00972	4,419326	3,54E-05	0,0018916	2,086426
ENSG00000177494	ZBED2	1,999749	1,5594952	4,445295	3,22E-05	0,0018097	2,235943
ENSG00000148848	ADAM12	1,979848	5,1276213	5,672007	2,94E-07	0,000102	6,51366
ENSG00000060718	COL11A1	1,950429	6,8878336	3,630713	0,000534	0,0107801	-0,65311
ENSG00000276170	LOC101929494	1,923438	0,1659096	4,668569	1,42E-05	0,0010534	2,898178
ENSG00000196549	MME	1,909035	3,7820566	4,094134	0,000112	0,0039318	0,971642
ENSG00000180447	GAS1	1,883241	4,2332225	6,456675	1,21E-08	2,17E-05	9,617899
ENSG00000016602	CLCA4	1,832308	-1,372113	3,668092	0,000473	0,0099306	-0,22662
ENSG00000163283	ALPP	1,829311	-0,929286	3,043129	0,003296	0,0349424	-1,80094
ENSG00000100985	MMP9	1,826966	3,894499	4,810414	8,37E-06	0,0007771	3,399823
ENSG00000265190	ANXA8	1,811216	2,1128948	4,413612	3,61E-05	0,0019197	2,127082
ENSG00000230838	NA	1,805318	1,5543935	3,883317	0,000231	0,0061757	0,456829
ENSG00000112319	EYA4	1,7958	0,7920694	4,50224	2,62E-05	0,0015833	2,40411
ENSG00000165323	FAT3	1,791561	0,6681884	4,300001	5,43E-05	0,0024276	1,750551
ENSG00000181374	CCL13	1,785642	2,0826886	3,848426	0,00026	0,0066133	0,330016
ENSG00000153993	SEMA3D	1,780984	1,7854012	4,747498	1,06E-05	0,0008956	3,250027
ENSG00000168334	XIRP1	1,779052	0,3059082	3,824935	0,000281	0,0069884	0,294941
ENSG00000134762	DSC3	1,767406	1,467753	3,363747	0,001251	0,0187323	-1,0468

ENSG00000134363	FST	1,765145	2,1628445	4,913355	5,67E-06	0,0006049	3,826541
ENSG00000203740	METTL11B	1,761021	-1,628656	4,220269	7,20E-05	0,0029044	1,277619
ENSG00000141579	ZNF750	1,755623	-0,960669	3,625664	0,000543	0,0108615	-0,31209
ENSG00000230333	NA	1,75473	0,4366488	4,861435	6,90E-06	0,0006917	3,561616
ENSG00000174564	IL20RB	1,752554	2,4285832	3,917074	0,000206	0,005747	0,522578
ENSG00000176177	ENTHD1	1,751611	-0,713936	3,964446	0,000175	0,0052303	0,652537
ENSG00000158270	COLEC12	1,741852	4,5206145	5,021719	3,76E-06	0,0004766	4,110816
ENSG00000204362	LOC400743	1,732205	-1,351354	3,46136	0,00092	0,0154633	-0,7693
ENSG00000122691	TWIST1	1,727525	2,8251016	5,263229	1,48E-06	0,0002777	5,07612
ENSG00000196754	S100A2	1,725928	4,401721	3,585925	0,000618	0,0119062	-0,68835
ENSG00000197632	SERPINB2	1,721487	3,0129161	2,9267	0,004618	0,0431579	-2,34188
ENSG00000157168	NRG1	1,717914	1,1153659	5,808729	1,70E-07	8,14E-05	6,959676
ENSG00000232679	NA	1,708009	0,6969081	4,793195	8,93E-06	0,0008123	3,357544
ENSG00000178776	C5orf46	1,694302	1,9824786	3,998687	0,000156	0,0048438	0,797556
ENSG00000131386	GALNT15	1,679585	2,631117	5,064822	3,18E-06	0,0004379	4,362024
ENSG00000073282	TP63	1,66532	1,5164444	3,20243	0,002052	0,0256209	-1,48696
ENSG00000108244	KRT23	1,662145	4,5411319	3,203948	0,002042	0,0255745	-1,80729
ENSG00000186832	KRT16	1,656164	4,9874913	3,620783	0,000552	0,0109623	-0,64211
ENSG00000136541	ERMN	1,645545	1,8410346	5,823656	1,60E-07	8,02E-05	7,09462
ENSG00000142549	IGLON5	1,644132	-0,114036	5,766337	2,02E-07	8,40E-05	6,561954
ENSG00000111799	COL12A1	1,643125	8,495642	5,610662	3,76E-07	0,0001232	6,302134
ENSG00000238042	LINC02257	1,63347	-0,075586	4,201915	7,69E-05	0,0030375	1,406024
ENSG00000086570	FAT2	1,632389	0,8888951	3,142183	0,002459	0,0289232	-1,60754
ENSG00000104055	TGM5	1,617433	-1,325487	2,93783	0,004473	0,0421764	-2,04916
ENSG00000251320	NA	1,616788	-0,612296	4,437222	3,32E-05	0,0018329	2,075221
ENSG00000124731	TREM1	1,606253	3,5170552	4,92799	5,37E-06	0,0005826	3,845136
ENSG00000115414	FN1	1,604506	11,301365	4,811305	8,34E-06	0,0007771	3,43485
ENSG00000198796	ALPK2	1,595838	2,2414045	4,737205	1,10E-05	0,0009033	3,21653
ENSG00000108821	COL1A1	1,595831	11,362477	4,853363	7,12E-06	0,0007009	3,585031
ENSG00000262406	MMP12	1,593677	3,8742238	4,293873	5,55E-05	0,0024566	1,620127
ENSG00000248323	LUCAT1	1,590068	0,8263434	4,277811	5,88E-05	0,0025306	1,685278
ENSG00000249992	TMEM158	1,589928	3,4429994	5,405474	8,47E-07	0,0002169	5,589118
ENSG00000243566	UPK3B	1,568954	1,1271454	3,705297	0,000418	0,0091327	-0,0616
ENSG00000064205	WISP2	1,563482	0,9581293	3,972141	0,000171	0,0051542	0,737467
ENSG00000244694	PTCHD4	1,551046	-0,780089	4,167187	8,68E-05	0,0033157	1,239242
ENSG00000147689	FAM83A	1,549826	4,3473824	3,007227	0,003661	0,0375631	-2,31552
ENSG00000162723	SLAMF9	1,548932	-0,946425	3,806644	0,000299	0,0072988	0,18634
ENSG00000171049	FPR2	1,548656	0,7783594	3,824366	0,000282	0,0069921	0,294972
ENSG00000167244	IGF2	1,542839	4,0465164	3,794913	0,000311	0,0074394	-0,00868
ENSG00000153976	HS3ST3A1	1,522181	0,6345102	4,424261	3,48E-05	0,0018805	2,144787
ENSG00000183876	ARSI	1,515221	2,2073482	4,984347	4,33E-06	0,0005169	4,075661
ENSG00000173267	SNCG	1,514726	2,7285569	4,279144	5,85E-05	0,002527	1,661897
ENSG00000145536	ADAMTS16	1,510204	1,1410566	3,751402	0,000359	0,008206	0,073384
ENSG00000183242	WT1-AS	1,50128	-0,890157	4,249278	6,50E-05	0,0027058	1,470453

ENSG00000124882	EREG	1,499067	3,562988	3,140346	0,002472	0,0290444	-1,85215
ENSG00000172137	CALB2	1,49868	2,6809838	3,539337	0,000718	0,0131576	-0,63181
ENSG00000113763	UNC5A	1,498171	0,4820336	3,625994	0,000542	0,0108615	-0,27763
ENSG00000134013	LOXL2	1,495955	6,1321694	5,870242	1,33E-07	6,83E-05	7,257719
ENSG00000147509	RGS20	1,487034	-0,726425	3,495698	0,000825	0,0143363	-0,65062
ENSG00000184937	WT1	1,478691	0,5369098	3,334611	0,001369	0,0197163	-1,08362
ENSG00000163220	S100A9	1,477464	5,4274044	4,080128	0,000118	0,0040549	0,778466
ENSG00000171388	APLN	1,477052	1,7138176	4,618512	1,71E-05	0,0012032	2,812497
ENSG00000154102	C16orf74	1,470271	1,497288	4,621318	1,69E-05	0,0012032	2,819104
ENSG00000124343	XG	1,465104	-0,680884	3,9014	0,000217	0,0059621	0,47386
ENSG00000167749	KLK4	1,461211	-1,09335	3,320153	0,001432	0,0202886	-1,11928
ENSG00000271216	LINC01050	1,46067	-0,411304	3,650251	0,000501	0,0103935	-0,21992
ENSG00000144057	ST6GAL2	1,453503	2,6490216	4,032742	0,000139	0,0044939	0,87179
ENSG00000164692	COL1A2	1,452029	11,489966	4,935485	5,22E-06	0,0005732	3,879827
ENSG00000090659	CD209	1,45147	2,3096395	4,649949	1,52E-05	0,0011148	2,91725
ENSG00000248362	NA	1,449838	-1,308662	3,832097	0,000274	0,0068439	0,225374
ENSG00000197614	MFAP5	1,444711	5,5406057	3,96881	0,000173	0,0051957	0,411471
ENSG00000176887	SOX11	1,442751	1,3732131	4,165698	8,73E-05	0,003319	1,335313
ENSG00000143320	CRABP2	1,437132	5,0791717	3,90241	0,000217	0,0059572	0,225826
ENSG00000166473	PKD1L2	1,425272	0,9589083	3,167155	0,002282	0,0274632	-1,54646
ENSG00000259225	LINC02345	1,423581	-0,490229	3,091811	0,002856	0,0319888	-1,68555
ENSG00000155269	GPR78	1,417512	-0,493146	3,597824	0,000594	0,0115929	-0,36707
ENSG00000231407	NA	1,414941	-0,022082	5,66496	3,03E-07	0,000103	6,231548
ENSG00000184985	SORCS2	1,398694	2,8265845	5,374018	9,59E-07	0,0002243	5,48076
ENSG00000183019	MCEMP1	1,38902	1,7668681	3,146379	0,002428	0,0286653	-1,6526
ENSG00000104213	PDGFRL	1,373593	3,6661224	4,44733	3,20E-05	0,0018058	2,157036
ENSG00000137699	TRIM29	1,370103	6,6914721	3,699133	0,000427	0,0092155	-0,44895
ENSG00000169429	CXCL8	1,368188	6,7794539	4,014023	0,000148	0,0046838	0,549707
ENSG00000166923	GREM1	1,365193	8,2200545	3,397257	0,001126	0,0175872	-1,28737
ENSG00000154027	AK5	1,360425	0,7313455	3,57398	0,000642	0,0122436	-0,42913
ENSG00000203805	PLPP4	1,358525	3,4402848	4,414793	3,60E-05	0,0019172	2,065998
ENSG00000165799	RNASE7	1,358108	-0,891533	2,850801	0,005726	0,0498269	-2,25207
ENSG00000258227	CLEC5A	1,357864	3,5991658	4,832208	7,71E-06	0,0007428	3,499068
ENSG00000160161	CILP2	1,356439	1,8635038	3,914402	0,000208	0,0057725	0,541332
ENSG00000143546	S100A8	1,354765	3,9119062	3,939499	0,000191	0,0054601	0,460889
ENSG00000186431	FCAR	1,353807	-0,584159	3,686279	0,000445	0,0094996	-0,12679
ENSG00000115008	IL1A	1,353271	2,0050123	3,140015	0,002475	0,0290542	-1,68733
ENSG00000142156	COL6A1	1,352456	8,1420414	5,325838	1,16E-06	0,000237	5,213793
ENSG00000088882	CPXM1	1,348144	4,2265131	4,286551	5,70E-05	0,0024901	1,559968
ENSG00000185640	KRT79	1,343294	-1,23532	3,277395	0,001633	0,0221252	-1,23167
ENSG00000222047	C10orf55	1,341809	0,8070231	4,562305	2,10E-05	0,0013625	2,600753
ENSG00000186407	CD300E	1,336846	1,7877041	4,551563	2,19E-05	0,0014021	2,588543
ENSG00000168779	SHOX2	1,333081	1,4102882	3,457265	0,000932	0,0155726	-0,78363
ENSG00000183671	GPR1	1,332072	0,7866642	3,827698	0,000279	0,0069335	0,304777

ENSG00000227066	NA	1,329508	2,0768234	2,927481	0,004607	0,043084	-2,24179
ENSG00000168542	COL3A1	1,32916	12,086567	4,940675	5,11E-06	0,0005725	3,909223
ENSG00000029559	IBSP	1,324298	0,3089919	3,083623	0,002926	0,0324299	-1,72838
ENSG00000137809	ITGA11	1,320665	5,8460956	4,457701	3,08E-05	0,0017747	2,03471
ENSG00000198759	EGFL6	1,318379	1,8469697	5,132862	2,45E-06	0,0003804	4,591036
ENSG00000140945	CDH13	1,313671	3,1208671	5,756597	2,10E-07	8,40E-05	6,907739
ENSG00000125726	CD70	1,312291	0,1728793	3,470625	0,000894	0,0151647	-0,70753
ENSG00000183638	RP1L1	1,304006	-0,27654	3,242866	0,001815	0,0236967	-1,30756
ENSG00000131355	ADGRE3	1,297196	-0,03411	3,942669	0,000189	0,0054446	0,630719
ENSG00000116132	PRRX1	1,296523	6,4243338	5,118101	2,59E-06	0,0003863	4,395967
ENSG00000059804	SLC2A3	1,291054	6,8338957	5,156916	2,23E-06	0,0003593	4,545678
ENSG00000271811	NA	1,279458	-1,294976	2,939082	0,004457	0,0421385	-2,04616
ENSG00000104415	WISP1	1,277602	5,0159158	5,506	5,70E-07	0,0001656	5,884268
ENSG00000134775	FHOD3	1,276043	3,0177534	4,111103	0,000106	0,0038236	1,097017
ENSG00000250771	LOC100419170	1,274832	-1,022018	3,489417	0,000842	0,0145157	-0,67891
ENSG00000105376	ICAM5	1,270929	0,4345592	3,317161	0,001445	0,0204056	-1,12704
ENSG00000122861	PLAU	1,267785	7,6164525	5,091544	2,87E-06	0,0004142	4,326317
ENSG00000073756	PTGS2	1,267423	5,7561872	3,483842	0,000857	0,0146703	-1,09653
ENSG00000142871	CYR61	1,260353	7,3100323	5,128623	2,49E-06	0,0003804	4,453451
ENSG00000087245	MMP2	1,258953	8,7104287	4,750496	1,05E-05	0,0008901	3,132658
ENSG00000103569	AQP9	1,236684	3,1239403	3,151887	0,002389	0,0283549	-1,76477
ENSG00000113083	LOX	1,233903	6,3443603	5,593432	4,03E-07	0,0001273	6,187035
ENSG00000136244	IL6	1,231162	1,8389923	3,183444	0,002173	0,0265978	-1,55944
ENSG00000259807	NA	1,231133	1,349839	4,240199	6,71E-05	0,0027389	1,571789
ENSG00000070808	CAMK2A	1,225559	0,1438075	4,106091	0,000108	0,003843	1,127412
ENSG00000041982	TNC	1,222574	5,7498645	2,944399	0,004389	0,0417607	-2,60024
ENSG00000142173	COL6A2	1,218507	8,4347953	4,890482	6,19E-06	0,0006482	3,622718
ENSG00000166250	CLMP	1,21764	5,0218978	4,558168	2,13E-05	0,0013734	2,420339
ENSG00000225614	ZNF469	1,215467	3,7303916	4,296174	5,50E-05	0,0024527	1,641971
ENSG00000123689	G0S2	1,215224	4,4840833	3,919433	0,000204	0,0057168	0,333685
ENSG00000204262	COL5A2	1,208143	9,1234504	5,000313	4,08E-06	0,0004993	4,045651
ENSG00000123610	TNFAIP6	1,20389	4,4159337	4,488767	2,75E-05	0,0016301	2,228742
ENSG00000164694	FNDC1	1,200759	5,5921763	3,385097	0,00117	0,0179276	-1,37935
ENSG00000223485	LINC01615	1,198468	-0,447076	4,011073	0,000149	0,0047233	0,808475
ENSG00000181458	TMEM45A	1,19282	5,4377049	4,180667	8,28E-05	0,0032031	1,109827
ENSG00000120708	TGFBI	1,181087	8,1285167	5,01541	3,85E-06	0,0004843	4,06468
ENSG00000172986	GXYLT2	1,177792	3,6016803	3,893923	0,000223	0,0060297	0,351687
ENSG00000278962	NA	1,175291	-0,288161	4,152924	9,13E-05	0,0034348	1,241892
ENSG00000184408	KCND2	1,17241	3,1889583	4,607625	1,78E-05	0,0012192	2,740848
ENSG00000105880	DLX5	1,171373	-0,18993	3,007593	0,003657	0,0375631	-1,89865
ENSG00000147697	GSDMC	1,171171	1,3275057	3,20873	0,002013	0,0253127	-1,45843
ENSG00000206538	VGLL3	1,167485	5,0610115	4,752279	1,04E-05	0,0008901	3,101859
ENSG00000099953	MMP11	1,165998	8,2469055	3,158389	0,002343	0,0279923	-1,95984
ENSG00000060982	BCAT1	1,165306	6,130672	3,78063	0,000326	0,0076499	-0,20066

ENSG00000034053	APBA2	1,164768	3,1423177	5,039991	3,50E-06	0,0004573	4,263708
ENSG00000130635	COL5A1	1,163005	8,7424319	4,328126	4,91E-05	0,0023163	1,670025
ENSG00000078098	FAP	1,160385	6,2856322	4,404343	3,73E-05	0,0019561	1,84676
ENSG00000145794	MEGF10	1,15872	0,0179129	2,879286	0,005284	0,0470239	-2,21555
ENSG00000110852	CLEC2B	1,155398	4,9239261	5,187583	1,98E-06	0,000334	4,693541
ENSG00000117069	ST6GALNAC5	1,153142	2,9327122	3,036281	0,003363	0,0353795	-2,0495
ENSG00000149596	JPH2	1,152466	2,2505559	2,95482	0,00426	0,041071	-2,18865
ENSG00000186340	THBS2	1,151909	9,1351825	4,027683	0,000141	0,0045404	0,69526
ENSG00000087494	PTHLH	1,15168	2,8221887	3,314357	0,001457	0,0204845	-1,2861
ENSG00000170558	CDH2	1,150614	3,1994658	3,564167	0,000663	0,0124918	-0,60878
ENSG00000262454	NA	1,149444	-0,006589	4,035646	0,000137	0,0044897	0,907883
ENSG00000188042	ARL4C	1,149052	7,2025736	5,252031	1,54E-06	0,0002839	4,908931
ENSG00000148344	PTGES	1,147348	4,7890158	4,642875	1,56E-05	0,0011352	2,733548
ENSG00000092969	TGFB2	1,139905	4,2266333	4,51167	2,53E-05	0,0015587	2,325205
ENSG00000130508	PXDN	1,138119	6,3597858	4,569159	2,05E-05	0,0013481	2,418032
ENSG00000117586	TNFSF4	1,126444	3,7525093	4,457352	3,08E-05	0,0017747	2,183394
ENSG00000274307	NA	1,119145	-0,168524	4,421611	3,51E-05	0,0018858	2,077025
ENSG00000128383	APOBEC3A	1,116212	-0,529189	3,005964	0,003674	0,0375935	-1,89425
ENSG00000179934	CCR8	1,112331	-0,409462	2,977233	0,003993	0,0396463	-1,96603
ENSG00000174332	GLIS1	1,111409	-0,929957	3,010952	0,003621	0,0373094	-1,87783
ENSG00000162493	PDPN	1,111034	5,5039648	4,953774	4,87E-06	0,000555	3,805643
ENSG00000099985	OSM	1,110458	3,353074	3,394502	0,001136	0,0176631	-1,11851
ENSG00000080573	COL5A3	1,107837	4,473133	4,762894	1,00E-05	0,0008746	3,182903
ENSG00000106366	SERPINE1	1,103956	7,4742158	3,424243	0,001035	0,016649	-1,24544
ENSG00000264230	ANXA8L1	1,103578	1,7658907	3,108192	0,002721	0,0309316	-1,75291
ENSG00000277632	CCL3	1,101512	1,8886596	3,861375	0,000249	0,0064589	0,378213
ENSG00000229056	HECW2-AS1	1,100411	1,4461819	4,347536	4,58E-05	0,0021953	1,916743
ENSG00000273340	NA	1,100288	0,0455803	3,560447	0,000671	0,0125868	-0,46025
ENSG00000103313	MEFV	1,098489	0,4571055	3,13741	0,002494	0,029205	-1,59806
ENSG00000087303	NID2	1,098193	4,0697837	4,390327	3,93E-05	0,0020108	1,925392
ENSG00000111728	ST8SIA1	1,097035	1,2716924	4,041557	0,000135	0,0044391	0,94809
ENSG00000148926	ADM	1,096324	5,5062884	3,073247	0,003017	0,0329602	-2,2478
ENSG00000134830	C5AR2	1,092255	1,9383541	4,88319	6,36E-06	0,0006586	3,718919
ENSG00000139926	FRMD6	1,090717	6,3937344	5,331656	1,13E-06	0,000237	5,192611
ENSG00000168621	GDNF	1,088742	-0,208048	3,245201	0,001802	0,0236315	-1,30223
ENSG00000163359	COL6A3	1,087947	9,9470088	4,280126	5,83E-05	0,002527	1,562131
ENSG00000205755	CRLF2	1,086672	-0,974902	3,112032	0,00269	0,0306805	-1,63455
ENSG00000253161	LINC01605	1,086175	-0,81662	3,166745	0,002285	0,0274632	-1,49906
ENSG00000171502	COL24A1	1,086079	2,578043	4,499319	2,65E-05	0,0015896	2,400484
ENSG00000124813	RUNX2	1,085244	4,9153989	6,122051	4,77E-08	3,58E-05	8,273903
ENSG00000272841	NA	1,084774	1,7510457	3,487072	0,000848	0,0145712	-0,71731
ENSG00000122641	INHBA	1,077751	7,6278517	4,392284	3,90E-05	0,002008	1,840248
ENSG00000143333	RGS16	1,073385	5,7091627	3,273391	0,001653	0,0222978	-1,70371
ENSG00000136859	ANGPTL2	1,07333	5,9633664	4,293852	5,55E-05	0,0024566	1,472747

ENSG00000161638	ITGA5	1,073236	6,8764466	5,226922	1,70E-06	0,0003008	4,806988
ENSG00000144810	COL8A1	1,072562	7,1797409	3,72378	0,000394	0,0087262	-0,35747
ENSG00000164932	CTHRC1	1,072203	7,4717069	4,070551	0,000122	0,0041366	0,757783
ENSG00000109705	NKX3-2	1,06957	1,750414	3,850627	0,000258	0,0066067	0,351007
ENSG00000175600	SUGCT	1,066898	3,5854303	3,594837	0,0006	0,0116551	-0,56196
ENSG00000144681	STAC	1,065316	1,7112019	2,889604	0,005132	0,0461937	-2,30512
ENSG00000261742	LINC00922	1,061807	-0,221214	3,032062	0,003405	0,0356729	-1,83791
ENSG00000057019	DCBLD2	1,061177	6,9756408	3,882569	0,000232	0,0061822	0,13309
ENSG00000233682	LOC101929122	1,060395	-0,70552	3,279712	0,001621	0,0219851	-1,21378
ENSG00000151388	ADAMTS12	1,056677	5,8434289	3,962056	0,000177	0,0052471	0,379562
ENSG00000131015	ULBP2	1,054297	0,952214	3,552412	0,000688	0,0127599	-0,49637
ENSG00000049249	TNFRSF9	1,050725	1,9770586	3,931832	0,000196	0,0055599	0,590498
ENSG00000132000	PODNL1	1,047757	3,0754176	3,92838	0,000198	0,0055909	0,511603
ENSG00000144152	FBLN7	1,045967	1,6209712	4,354751	4,46E-05	0,002181	1,939925
ENSG00000176170	SPHK1	1,045438	5,1661658	4,364964	4,30E-05	0,0021265	1,745189
ENSG00000171812	COL8A2	1,045184	5,216297	3,63136	0,000533	0,0107713	-0,62736
ENSG00000101463	SYNDIG1	1,04277	2,8790213	5,072606	3,09E-06	0,0004316	4,387238
ENSG00000082196	C1QTNF3	1,04127	4,9955051	3,251655	0,001767	0,0233194	-1,72087
ENSG00000257894	NA	1,039167	-0,34334	3,394682	0,001135	0,0176631	-0,91141
ENSG00000105974	CAV1	1,039142	6,7289563	5,133149	2,45E-06	0,0003804	4,455544
ENSG00000167123	CERCAM	1,036834	6,1191642	4,751732	1,04E-05	0,0008901	3,063646
ENSG00000275832	ARHGAP23	1,036232	5,0455013	4,24468	6,61E-05	0,0027127	1,346909
ENSG00000106624	AEBP1	1,035996	8,6605687	3,778777	0,000328	0,0076673	-0,11808
ENSG00000143226	FCGR2A	1,03497	5,6165932	4,437387	3,31E-05	0,0018329	1,971207
ENSG00000137727	ARHGAP20	1,033627	2,154543	4,008544	0,000151	0,0047396	0,821402
ENSG00000272211	NA	1,032984	0,436181	3,485906	0,000851	0,0146114	-0,66911
ENSG00000166073	GPR176	1,030857	3,7106076	5,05687	3,28E-06	0,0004393	4,297165
ENSG00000260314	MRC1	1,028423	5,7944561	2,994888	0,003794	0,0383466	-2,46899
ENSG00000102802	MEDAG	1,027879	3,8279729	3,081012	0,002949	0,0325408	-2,04836
ENSG00000261327	NA	1,022088	0,5436513	3,270021	0,00167	0,0224619	-1,25605
ENSG00000106571	GLI3	1,021849	3,5924535	4,038263	0,000136	0,0044658	0,811112
ENSG00000105609	LILRB5	1,018916	2,279043	3,415803	0,001063	0,0169756	-0,95383
ENSG00000128274	A4GALT	1,017716	4,3609878	4,136789	9,66E-05	0,0036043	1,049343
ENSG00000123500	COL10A1	1,016813	7,9423448	3,395761	0,001132	0,0176393	-1,30592
ENSG00000147614	ATP6V0D2	1,016755	0,2450908	2,887318	0,005165	0,0463919	-2,20787
ENSG00000165474	GJB2	1,016326	6,713587	4,431877	3,38E-05	0,0018466	1,94628
ENSG00000018280	SLC11A1	1,015702	5,429377	3,462962	0,000916	0,0154215	-1,14405
ENSG00000123496	IL13RA2	1,01559	-0,486209	3,469093	0,000898	0,0152243	-0,71531
ENSG00000145423	SFRP2	1,015318	8,6373502	2,947236	0,004354	0,0415436	-2,50231
ENSG00000177234	LINC01561	1,012575	-0,414379	3,234645	0,001861	0,0241027	-1,32774
ENSG00000143387	CTSK	1,011853	7,9046222	3,781109	0,000326	0,0076477	-0,14809
ENSG00000175906	ARL4D	1,01178	2,9003159	3,417349	0,001057	0,0169534	-1,00358
ENSG00000170891	CYTL1	1,011278	-0,030218	3,014247	0,003587	0,0370591	-1,88767
ENSG00000180509	KCNE1	1,00339	-0,685856	3,082956	0,002932	0,0324449	-1,70538
ENSG00000197646	PDCD1LG2	1,003375	1,9911298	4,022574	0,000144	0,0045882	0,871816

Metabolic Cluster

Ensmbl_ID	Gene	logFC	AveExpr	t	P.Value	adj.P.Val	B
ENSG00000134193	REG4	4,023635	5,1999608	5,198102	1,90E-06	0,0001027	4,72568
ENSG00000090402	SI	4,016278	-1,340839	4,880964	6,41E-06	0,0002231	3,300946
ENSG00000169876	MUC17	3,985688	3,9429295	5,083626	2,96E-06	0,0001345	4,383053
ENSG00000163586	FABP1	3,806968	-1,05763	4,939736	5,13E-06	0,0001956	3,553743
ENSG00000016490	CLCA1	3,800585	-0,757857	4,876677	6,52E-06	0,0002263	3,418239
ENSG00000113303	BTNL8	3,772505	1,9172621	7,856076	3,38E-11	2,03E-07	14,85192
ENSG00000168955	TM4SF20	3,539458	0,8081089	4,361572	4,36E-05	0,0008368	1,948501
ENSG00000174358	SLC6A19	3,52218	0,3239713	5,760151	2,07E-07	2,54E-05	6,627769
ENSG00000171431	KRT20	3,340727	0,7125891	3,523649	0,000755	0,006707	-0,56852
ENSG00000166869	CHP2	3,310911	-1,408071	4,189272	8,04E-05	0,0012989	1,211023
ENSG00000112818	MEP1A	3,273343	-1,391986	5,097871	2,80E-06	0,0001319	3,966783
ENSG00000134240	HMGCS2	3,261628	2,1287372	4,339753	4,71E-05	0,0008886	1,885478
ENSG00000187908	DMBT1	3,214819	6,3506432	5,261142	1,49E-06	8,55E-05	4,938599
ENSG00000079112	CDH17	3,149816	4,2511204	6,040365	6,66E-08	1,25E-05	7,982463
ENSG00000263429	TMEM238L	3,099581	0,8167287	5,639778	3,35E-07	3,43E-05	6,29462
ENSG00000233041	PHGR1	2,98948	0,6203836	5,140702	2,38E-06	0,000118	4,519472
ENSG00000144820	ADGRG7	2,905869	0,362106	4,144895	9,39E-05	0,0014439	1,252366
ENSG00000166391	MOGAT2	2,879013	0,2454929	4,669022	1,42E-05	0,0003831	2,900186
ENSG00000163501	IHH	2,854408	2,9249398	6,297302	2,33E-08	6,55E-06	8,963338
ENSG00000167117	ANKRD40CL	2,843705	-0,178717	4,917074	5,59E-06	0,0002074	3,653835
ENSG00000243766	NA	2,831329	0,0086421	4,042105	0,000134	0,0018595	0,92405
ENSG00000166866	MYO1A	2,817606	2,856402	5,935173	1,02E-07	1,57E-05	7,574426
ENSG00000248771	SMIM31	2,814468	2,0472876	6,828335	2,57E-09	1,70E-06	10,90985
ENSG00000066230	SLC9A3	2,757952	2,4007761	5,373057	9,62E-07	6,35E-05	5,468399
ENSG00000168903	BTNL3	2,757579	-1,502969	3,8629	0,000247	0,0029129	0,280119
ENSG00000132437	DDC	2,698364	1,9347305	5,802241	1,75E-07	2,28E-05	7,016069
ENSG00000007216	SLC13A2	2,690014	-1,02149	4,390166	3,93E-05	0,000774	1,862641
ENSG00000179914	ITLN1	2,607902	0,0576538	3,899166	0,000219	0,0026711	0,503625
ENSG00000197408	CYP2B6	2,593376	-0,606105	4,474077	2,90E-05	0,0006224	2,17919
ENSG00000169994	MYO7B	2,548498	3,9052014	5,2429	1,60E-06	9,07E-05	4,969109
ENSG00000165556	CDX2	2,532655	1,485962	6,164309	4,02E-08	9,62E-06	8,309065
ENSG00000180745	CLRN3	2,524949	2,1657827	5,167358	2,14E-06	0,0001111	4,721081
ENSG00000143167	GPA33	2,508619	1,8071299	5,029042	3,65E-06	0,0001571	4,22126
ENSG00000060566	CREB3L3	2,484844	-0,627673	4,741066	1,08E-05	0,0003199	3,011165
ENSG00000178597	PSAPL1	2,484028	0,1107074	4,121811	0,000102	0,0015224	1,169429
ENSG00000106384	MOGAT3	2,481708	-0,521592	3,801638	0,000304	0,0034065	0,195338
ENSG00000066405	CLDN18	2,467099	7,2044155	3,191149	0,002123	0,0140947	-1,90319
ENSG00000237070	LOC105375166	2,459218	0,9396808	4,064953	0,000124	0,0017657	1,021286
ENSG00000274993	LOC105375431	2,453116	0,1827145	5,364857	9,94E-07	6,48E-05	5,220991
ENSG00000070019	GUCY2C	2,433526	0,9624441	5,398134	8,72E-07	5,97E-05	5,457431
ENSG00000198074	AKR1B10	2,38166	4,5635109	5,236579	1,64E-06	9,24E-05	4,904076
ENSG00000114248	LRRC31	2,35801	1,5703473	4,77706	9,48E-06	0,0002891	3,344282

ENSG00000226812	LOC105372629	2,337524	-0,822734	5,685419	2,79E-07	3,03E-05	6,056426
ENSG00000173702	MUC13	2,317073	6,6951633	4,807693	8,45E-06	0,0002682	3,280548
ENSG00000104537	ANXA13	2,29835	2,206871	4,711965	1,21E-05	0,0003425	3,13046
ENSG00000175311	ANKS4B	2,280989	2,6919735	5,415273	8,15E-07	5,84E-05	5,629628
ENSG00000142484	TM4SF5	2,245657	1,6476407	4,798398	8,75E-06	0,0002762	3,419132
ENSG00000229719	MIR194-2HG	2,237242	2,3857923	5,745973	2,19E-07	2,57E-05	6,839727
ENSG00000084674	APOB	2,203211	-0,371698	3,299989	0,001524	0,0110791	-1,15979
ENSG00000166959	MS4A8	2,195001	2,7714784	5,418189	8,06E-07	5,83E-05	5,641603
ENSG00000248144	ADH1C	2,183026	4,4685279	4,784258	9,23E-06	0,0002838	3,265719
ENSG00000144852	NR1I2	2,176215	3,6403253	4,640726	1,57E-05	0,0004131	2,828259
ENSG00000271824	SMIM32	2,171267	0,0351582	5,193821	1,94E-06	0,0001035	4,611301
ENSG00000172367	PDZD3	2,166608	2,4457587	4,338942	4,72E-05	0,0008886	1,873878
ENSG00000107807	TLX1	2,162835	-1,722108	4,27529	5,93E-05	0,0010464	1,404239
ENSG00000002726	AOC1	2,146165	5,0674819	6,959271	1,49E-09	1,32E-06	11,62025
ENSG00000160868	CYP3A4	2,139663	-0,605502	4,875007	6,56E-06	0,0002265	3,442529
ENSG00000107242	PIP5K1B	2,127124	3,7827827	8,12999	1,06E-11	9,52E-08	16,32154
ENSG00000188993	LRRC66	2,092319	2,6641091	6,014188	7,41E-08	1,30E-05	7,863896
ENSG00000278484	NA	2,091629	-1,105418	3,712973	0,000408	0,004256	-0,09039
ENSG00000249395	CASC9	2,091146	-0,150363	3,029399	0,003432	0,0198835	-1,8443
ENSG00000204876	LOC389602	2,082829	2,8662496	5,623013	3,58E-07	3,54E-05	6,40102
ENSG00000130234	ACE2	2,080409	2,9504397	4,587569	1,92E-05	0,0004703	2,687671
ENSG00000095932	SMIM24	2,066778	3,7739985	5,455585	6,95E-07	5,40E-05	5,765785
ENSG00000127831	VIL1	2,060836	4,5893522	3,77747	0,000329	0,0036204	-0,1147
ENSG00000009765	IYD	2,05953	3,6678363	4,757425	1,02E-05	0,0003055	3,234079
ENSG00000148584	A1CF	2,05194	2,2439739	4,925897	5,41E-06	0,0002035	3,870292
ENSG00000173597	SULT1B1	2,043859	4,7784206	4,154009	9,10E-05	0,0014094	1,075342
ENSG00000106031	HOXA13	2,019125	1,8160325	2,917639	0,004739	0,0251004	-2,23706
ENSG00000205277	MUC12	2,019061	1,6099969	4,627594	1,65E-05	0,0004243	2,840515
ENSG00000111701	APOBEC1	2,017369	1,2252158	4,594295	1,87E-05	0,0004633	2,720149
ENSG00000257743	MGAM2	2,016264	3,8897889	3,620431	0,000552	0,0052865	-0,51553
ENSG00000099834	CDHR5	1,995203	4,1817272	5,473713	6,47E-07	5,16E-05	5,813707
ENSG00000171747	LGALS4	1,991272	7,9729721	4,581172	1,96E-05	0,0004776	2,51988
ENSG00000240602	AADACP1	1,960178	1,3166838	3,530147	0,000739	0,0066113	-0,56993
ENSG00000173237	C11orf86	1,937766	0,3571465	3,719987	0,000399	0,0041841	-0,00919
ENSG00000168907	PLA2G4F	1,92997	1,5858609	5,906351	1,15E-07	1,71E-05	7,365861
ENSG00000062524	LTK	1,909263	1,7410132	5,672721	2,94E-07	3,17E-05	6,523102
ENSG00000188833	ENTPD8	1,901844	2,8311753	6,752954	3,52E-09	2,11E-06	10,7241
ENSG00000249853	HS3ST5	1,89946	-0,95599	4,020765	0,000145	0,0019707	0,784765
ENSG00000100433	KCNK10	1,88925	-0,138478	4,564637	2,08E-05	0,0005001	2,522465
ENSG00000169894	MUC3A	1,886283	6,7404439	4,625835	1,66E-05	0,0004264	2,635369
ENSG00000131910	NR0B2	1,880387	1,5324993	3,694115	0,000434	0,0044489	-0,10485
ENSG00000204616	TRIM31	1,87068	5,1715446	5,117227	2,60E-06	0,0001251	4,427719
ENSG00000275718	CCL15	1,86658	-0,576322	4,147344	9,31E-05	0,0014353	1,193176
ENSG00000116771	AGMAT	1,845907	1,8944949	7,296439	3,60E-10	5,90E-07	12,68422

ENSG00000229970	LOC100505938	1,8452	-1,581087	3,133307	0,002525	0,0158928	-1,59847
ENSG00000069764	PLA2G10	1,836961	2,1186812	5,236555	1,64E-06	9,24E-05	4,966686
ENSG00000187288	CIDEC	1,833785	0,2469022	3,418406	0,001054	0,008517	-0,84946
ENSG00000168350	DEGS2	1,831316	3,0469458	7,36149	2,74E-10	5,48E-07	13,14578
ENSG00000160180	TFF3	1,830599	5,5257041	3,224309	0,00192	0,0130764	-1,82026
ENSG00000162989	KCNJ3	1,829386	0,8402217	3,959815	0,000178	0,002291	0,70008
ENSG00000255774	NA	1,828482	-1,094889	4,592578	1,88E-05	0,0004643	2,46419
ENSG00000166268	MYRFL	1,826845	-0,436577	4,348443	4,57E-05	0,000867	1,814215
ENSG00000099617	EFNA2	1,817253	-0,064944	4,796003	8,83E-06	0,0002768	3,275528
ENSG00000151715	TMEM45B	1,814431	5,4603494	6,380422	1,65E-08	5,05E-06	9,286203
ENSG00000186603	HPDL	1,813215	0,7999932	5,491693	6,03E-07	4,96E-05	5,766295
ENSG00000165828	PRAP1	1,810019	-1,109147	3,222247	0,001932	0,0131388	-1,36752
ENSG00000159184	HOXB13	1,781106	-0,899954	2,651023	0,009918	0,0425118	-2,69354
ENSG00000075073	TACR2	1,772544	-0,245513	4,120241	0,000102	0,0015282	1,141158
ENSG00000164749	HNF4G	1,761112	4,7080588	6,320889	2,11E-08	6,09E-06	9,069718
ENSG00000136872	ALDOB	1,760293	4,0659711	3,15358	0,002377	0,0152306	-1,87682
ENSG00000101076	HNF4A	1,748154	5,40859	5,006753	3,98E-06	0,0001659	4,011971
ENSG00000249948	GBA3	1,725908	-0,213716	3,439969	0,000985	0,0081328	-0,79047
ENSG00000198203	SULT1C2	1,715069	6,0875344	3,745682	0,000366	0,0039185	-0,29482
ENSG00000164690	SHH	1,713583	3,3660492	5,030187	3,64E-06	0,0001567	4,222072
ENSG00000121380	BCL2L14	1,69461	3,4461082	8,748478	7,69E-13	1,39E-08	18,74493
ENSG00000274979	NA	1,692068	1,4799161	3,968653	0,000173	0,0022432	0,721398
ENSG00000198643	FAM3D	1,691135	4,0623017	3,482202	0,000861	0,0073854	-0,94671
ENSG00000266010	GATA6-AS1	1,688654	2,7947745	5,45865	6,87E-07	5,38E-05	5,790714
ENSG00000100170	SLC5A1	1,688072	5,1730418	5,421998	7,94E-07	5,83E-05	5,565623
ENSG00000124253	PCK1	1,687653	1,3788794	2,90282	0,004943	0,0258936	-2,2401
ENSG00000215018	COL28A1	1,686156	2,1879027	4,499028	2,65E-05	0,0005836	2,410903
ENSG00000224511	LINC00365	1,683012	-0,513071	4,967584	4,62E-06	0,0001813	3,759236
ENSG00000198758	EPS8L3	1,675254	6,0756098	4,019551	0,000145	0,0019751	0,575387
ENSG00000211454	AKR7L	1,673648	2,2767855	5,660779	3,08E-07	3,23E-05	6,517616
ENSG00000184434	LRRC19	1,666807	1,2161577	3,794021	0,000312	0,0034705	0,199592
ENSG00000047597	XK	1,658584	2,9578893	5,81817	1,64E-07	2,20E-05	7,135509
ENSG00000204128	C2orf72	1,627648	4,0133513	5,499614	5,84E-07	4,93E-05	5,919997
ENSG00000172955	ADH6	1,625197	1,4060051	4,325659	4,95E-05	0,000924	1,845577
ENSG00000118322	ATP10B	1,621029	5,2559335	4,30478	5,34E-05	0,0009676	1,546091
ENSG00000021826	CPS1	1,617317	3,2684854	3,098612	0,002799	0,0171232	-1,91813
ENSG00000139515	PDX1	1,616695	2,776073	3,987857	0,000162	0,0021377	0,726359
ENSG00000126562	WNK4	1,616157	0,2191018	3,988811	0,000161	0,0021355	0,774616
ENSG00000073067	CYP2W1	1,614297	0,8722575	3,72752	0,000389	0,004112	0,010404
ENSG00000139865	TTC6	1,607549	-0,136671	4,091333	0,000113	0,0016504	1,062424
ENSG00000258837	NA	1,604003	-1,622527	2,966804	0,004115	0,0226301	-1,98765
ENSG00000213214	ARHGEF35	1,599667	1,1869218	3,825199	0,000281	0,0032141	0,292967
ENSG00000241635	UGT1A1	1,58778	1,1426709	3,920663	0,000204	0,0025277	0,580429
ENSG00000189325	C6orf222	1,578346	4,4025225	3,35356	0,001291	0,0098356	-1,36033

ENSG00000137825	ITPKA	1,571638	2,0353328	4,456473	3,09E-05	0,0006523	2,271326
ENSG00000197520	FAM177B	1,56675	1,3497093	3,226628	0,001907	0,0130045	-1,40713
ENSG00000175538	KCNE3	1,562931	5,9214443	6,033682	6,84E-08	1,25E-05	7,906849
ENSG00000279693	NA	1,562337	0,0398423	4,982067	4,37E-06	0,0001752	3,902765
ENSG00000272189	NA	1,56176	0,197204	6,04388	6,57E-08	1,25E-05	7,602724
ENSG00000106541	AGR2	1,554088	9,2806017	5,728668	2,35E-07	2,68E-05	6,779934
ENSG00000086696	HSD17B2	1,553292	5,2471929	4,953214	4,88E-06	0,0001886	3,82367
ENSG00000173467	AGR3	1,550535	5,2089643	4,95589	4,83E-06	0,0001875	3,835195
ENSG00000111863	ADTRP	1,54839	3,2899181	4,738131	1,10E-05	0,0003219	3,190704
ENSG00000170231	FABP6	1,546044	-0,123292	2,78051	0,006967	0,0328293	-2,43601
ENSG00000186854	TRABD2A	1,54491	3,1647685	4,552094	2,18E-05	0,0005161	2,555408
ENSG00000167600	CYP2S1	1,537855	5,4582726	4,596017	1,86E-05	0,0004629	2,539044
ENSG00000176153	GPX2	1,534719	7,4539433	4,614908	1,73E-05	0,0004377	2,618546
ENSG00000139540	SLC39A5	1,52895	2,2642884	2,789713	0,006792	0,0322003	-2,59211
ENSG00000198099	ADH4	1,50873	-1,266216	2,663828	0,009582	0,0415172	-2,65662
ENSG00000127324	TSPAN8	1,505244	8,8095592	3,755737	0,000354	0,0038241	-0,16633
ENSG00000112280	COL9A1	1,503675	0,5815853	3,365421	0,001244	0,0096257	-0,9998
ENSG00000128610	FEZF1	1,497145	-1,267005	2,74603	0,007662	0,0352323	-2,47924
ENSG00000222001	LOC101928881	1,494752	0,3949043	4,096911	0,000111	0,0016239	1,107132
ENSG00000256612	CYP2B7P	1,494091	1,4462109	3,284422	0,001598	0,0114557	-1,25743
ENSG00000248596	LOC643201	1,493932	-1,413599	3,658618	0,000488	0,0048532	-0,26246
ENSG00000178828	RNF186	1,486213	0,5064887	3,077447	0,00298	0,0179366	-1,74957
ENSG00000188959	C9orf152	1,485426	3,9863316	4,4351	3,34E-05	0,0006903	2,091363
ENSG00000265763	ZNF488	1,476333	1,7577677	4,551459	2,19E-05	0,0005166	2,587727
ENSG00000249923	LOC284865	1,472461	-0,01028	2,684459	0,009063	0,0397455	-2,65981
ENSG00000167183	PRR15L	1,468145	6,1297017	5,175376	2,08E-06	0,0001087	4,618763
ENSG00000118513	MYB	1,462334	2,1025435	5,333984	1,12E-06	7,04E-05	5,315983
ENSG00000173890	GPR160	1,460742	5,0349095	7,527286	1,36E-10	3,50E-07	13,93168
ENSG00000247844	NA	1,458242	1,6683435	3,133457	0,002524	0,0158913	-1,67364
ENSG00000140297	GCNT3	1,45714	7,2443725	4,128243	9,95E-05	0,001501	0,9535
ENSG00000019102	VSIG2	1,45266	5,9045591	3,294282	0,001551	0,0112075	-1,63534
ENSG00000123191	ATP7B	1,450747	4,0203705	4,92294	5,47E-06	0,0002046	3,796465
ENSG00000166126	AMN	1,441346	4,1955809	4,88358	6,35E-06	0,0002228	3,641873
ENSG00000124102	PI3	1,437476	3,8492275	3,063062	0,003109	0,0185765	-2,09117
ENSG00000124429	POF1B	1,433859	6,2499436	6,382368	1,64E-08	5,05E-06	9,28653
ENSG00000251637	LOC105369360	1,430163	-0,960969	4,635568	1,61E-05	0,0004177	2,621369
ENSG00000203727	SAMD5	1,424288	4,1971882	5,070642	3,11E-06	0,0001403	4,31825
ENSG00000170608	FOXA3	1,418344	4,6004821	4,588817	1,91E-05	0,0004693	2,566729
ENSG00000243955	GSTA1	1,416638	3,1599687	2,952505	0,004288	0,0233179	-2,2866
ENSG00000137968	SLC44A5	1,413494	2,6775103	2,720952	0,008207	0,0371024	-2,80204
ENSG00000144045	DQX1	1,402643	2,5835006	2,618549	0,010818	0,04533	-3,03212
ENSG00000104371	DKK4	1,398187	-0,796735	3,154213	0,002372	0,015215	-1,53046
ENSG00000237223	SULT1C2P1	1,389938	-0,363806	3,119443	0,002632	0,0163521	-1,61779
ENSG00000131771	PPP1R1B	1,388955	5,7350192	3,835751	0,000271	0,0031282	-0,00686

ENSG00000257335	MGAM	1,385194	1,3325217	4,42019	3,53E-05	0,0007214	2,150946
ENSG00000134716	CYP2J2	1,385118	2,9253323	4,858197	6,99E-06	0,000235	3,626585
ENSG00000162779	AXDND1	1,380107	-0,087969	3,069237	0,003053	0,0182905	-1,74788
ENSG00000178750	STX19	1,372196	1,8073747	4,753105	1,04E-05	0,0003079	3,268109
ENSG00000168453	HR	1,36829	3,6662617	6,421685	1,39E-08	4,65E-06	9,479834
ENSG00000188242	PP7080	1,366831	3,6947344	5,944722	9,82E-08	1,53E-05	7,625103
ENSG00000164120	HPGD	1,361238	5,7629748	4,231015	6,94E-05	0,0011693	1,277962
ENSG00000170439	METTL7B	1,35521	2,9198347	4,460721	3,05E-05	0,0006449	2,259006
ENSG00000168743	NPNT	1,355076	6,4273563	4,6471	1,54E-05	0,0004058	2,705226
ENSG00000150750	C11orf53	1,351106	0,3806818	3,169063	0,002269	0,0147113	-1,51074
ENSG00000113722	CDX1	1,350939	0,8802137	2,650535	0,009931	0,0425474	-2,79915
ENSG00000241388	HNF1A-AS1	1,350689	2,7325993	3,142555	0,002456	0,0156017	-1,73927
ENSG00000268388	FENDRR	1,350586	2,6751135	4,169781	8,61E-05	0,0013586	1,312456
ENSG00000198944	SOWAHA	1,340449	1,8679619	4,883181	6,36E-06	0,0002228	3,715825
ENSG00000135100	HNF1A	1,339576	2,0853943	4,636696	1,60E-05	0,0004168	2,874671
ENSG00000173557	C2orf70	1,338486	0,996017	4,690982	1,31E-05	0,0003596	3,031361
ENSG00000144354	CDCA7	1,334817	4,9465582	5,038964	3,52E-06	0,0001534	4,152158
ENSG00000260196	NA	1,331591	2,1035275	5,151052	2,28E-06	0,0001143	4,661231
ENSG00000267296	CEBPA-DT	1,328006	0,4703238	3,877131	0,000236	0,0028131	0,449598
ENSG00000196188	CTSE	1,323939	9,0824375	2,893435	0,005077	0,0264016	-2,5965
ENSG00000204610	TRIM15	1,323634	3,6528684	3,764297	0,000344	0,0037415	-0,0505
ENSG00000229155	LINC02038	1,320144	0,0981375	3,543511	0,000708	0,0063846	-0,50715
ENSG00000180061	TMEM150B	1,314931	1,0272985	3,485568	0,000852	0,0073452	-0,68441
ENSG00000171433	GLOD5	1,312148	-0,842966	4,165048	8,75E-05	0,0013748	1,216994
ENSG00000162482	AKR7A3	1,31166	4,1006988	4,090675	0,000113	0,0016528	0,932838
ENSG00000187699	C2orf88	1,309165	3,5637966	4,896321	6,05E-06	0,0002172	3,732647
ENSG00000261762	NA	1,308101	0,1812673	4,24005	6,72E-05	0,0011442	1,535199
ENSG00000099960	SLC7A4	1,304391	1,1167863	3,651529	0,000499	0,0049324	-0,21538
ENSG00000109255	NMU	1,304286	2,5158757	3,224814	0,001917	0,0130615	-1,49656
ENSG00000178538	CA8	1,298362	2,7485107	3,860517	0,000249	0,0029307	0,331554
ENSG00000188211	NCR3LG1	1,29674	3,6434544	5,520139	5,39E-07	4,76E-05	6,012407
ENSG00000187210	GCNT1	1,295389	5,4083512	5,724535	2,39E-07	2,70E-05	6,712576
ENSG00000130957	FBP2	1,288595	-1,65387	2,735006	0,007898	0,0360552	-2,50065
ENSG00000132744	ACY3	1,288592	2,1181167	4,632406	1,62E-05	0,0004193	2,860047
ENSG00000176532	PRR15	1,287866	4,5185211	6,9405	1,61E-09	1,32E-06	11,55033
ENSG00000129514	FOXA1	1,287835	3,0990511	3,588016	0,000614	0,0057175	-0,52172
ENSG00000101333	PLCB4	1,287223	4,5459902	4,777883	9,45E-06	0,0002888	3,237018
ENSG00000128298	BAIAP2L2	1,285514	4,4816121	3,472423	0,000889	0,0075644	-1,02503
ENSG00000095539	SEMA4G	1,281656	5,0585719	5,151445	2,28E-06	0,0001143	4,559657
ENSG00000164879	CA3	1,279217	1,4783202	5,174308	2,09E-06	0,0001087	4,71481
ENSG00000167588	GPD1	1,275202	2,4576304	3,032369	0,003402	0,0197689	-2,00215
ENSG00000170915	PAQR8	1,272294	5,182073	6,198871	3,49E-08	8,97E-06	8,572505
ENSG00000165810	BTNL9	1,267148	3,0666198	4,242955	6,65E-05	0,0011368	1,52656
ENSG00000106258	CYP3A5	1,263799	7,6142962	3,939449	0,000191	0,0024161	0,354906

ENSG00000103460	TOX3	1,262621	4,1806341	4,934432	5,24E-06	0,0001983	3,825739
ENSG00000260704	LINC00543	1,261436	0,0525127	3,690555	0,000439	0,0044897	-0,09696
ENSG00000104267	CA2	1,261361	5,5232403	4,11503	0,000104	0,0015484	0,900536
ENSG00000181378	CFAP65	1,259792	-0,059308	3,521082	0,000761	0,0067524	-0,56974
ENSG00000163293	NIPAL1	1,258374	3,4234623	6,422045	1,39E-08	4,65E-06	9,474143
ENSG00000213160	KLHL23	1,255982	4,7646351	7,772658	4,81E-11	2,17E-07	14,93193
ENSG00000140284	SLC27A2	1,251769	2,3182506	4,539905	2,28E-05	0,0005292	2,54574
ENSG00000138109	CYP2C9	1,251697	3,2062567	2,838701	0,005925	0,0292573	-2,58041
ENSG00000256340	ABCC6P1	1,24941	-1,084651	3,036752	0,003359	0,019612	-1,81696
ENSG00000262188	LINC01978	1,246645	1,3405062	4,142696	9,46E-05	0,0014532	1,263506
ENSG00000072954	TMEM38A	1,244626	3,3374691	4,88241	6,38E-06	0,0002228	3,695809
ENSG00000108352	RAPGEFL1	1,238513	5,676548	4,533739	2,33E-05	0,0005363	2,314186
ENSG00000087085	ACHE	1,236177	4,0014836	3,393306	0,00114	0,0090166	-1,19693
ENSG00000137960	GIPC2	1,232545	4,2842965	5,480285	6,31E-07	5,10E-05	5,832781
ENSG00000062282	DGAT2	1,230658	3,088172	4,837731	7,55E-06	0,0002465	3,549167
ENSG00000229167	NA	1,230275	0,1860231	4,231669	6,92E-05	0,0011677	1,509654
ENSG00000006611	USH1C	1,229144	5,8044067	3,669125	0,000471	0,0047415	-0,52643
ENSG00000170703	TTLL6	1,226452	0,9657216	2,799991	0,006601	0,0316519	-2,4585
ENSG00000108242	CYP2C18	1,225309	3,4641156	3,484501	0,000855	0,0073526	-0,86564
ENSG00000233930	KRTAP5-AS1	1,223098	0,0909979	4,629073	1,64E-05	0,0004232	2,754509
ENSG00000123843	C4BPB	1,220307	3,8926662	3,709892	0,000412	0,0042843	-0,24507
ENSG00000176387	HSD11B2	1,219724	3,8537042	4,874202	6,58E-06	0,0002267	3,634299
ENSG00000139629	GALNT6	1,218342	5,0519131	5,498159	5,88E-07	4,93E-05	5,859399
ENSG00000136574	GATA4	1,217341	3,6267196	3,528927	0,000742	0,0066273	-0,75533
ENSG00000137757	CASP5	1,215925	-0,771714	3,387244	0,001162	0,0091672	-0,93978
ENSG00000163817	SLC6A20	1,213064	5,3636468	4,029028	0,00014	0,0019232	0,626473
ENSG00000255468	LOC102724064	1,212097	0,9167002	4,780227	9,37E-06	0,0002872	3,32464
ENSG00000262772	LINC01977	1,210318	-1,066542	3,011489	0,003616	0,0206573	-1,87666
ENSG00000205089	CCNI2	1,20645	1,7640456	5,399105	8,69E-07	5,97E-05	5,532353
ENSG00000186529	CYP4F3	1,19736	4,0981866	4,124467	0,000101	0,0015133	1,043308
ENSG00000160867	FGFR4	1,186819	4,527988	4,031267	0,000139	0,0019157	0,696524
ENSG00000186204	CYP4F12	1,186597	4,0878572	4,03213	0,000139	0,0019157	0,74457
ENSG00000254166	CCAT1	1,185983	-1,421927	2,874137	0,005362	0,0273815	-2,19483
ENSG00000203943	SAMD13	1,185034	1,8697208	4,977276	4,45E-06	0,000177	4,04251
ENSG00000188611	ASAH2	1,184822	-0,003865	3,554321	0,000684	0,0062317	-0,47804
ENSG00000141052	MYOCD	1,184688	0,5442941	2,901359	0,004964	0,0259695	-2,18696
ENSG00000106404	CLDN15	1,180315	3,5262467	4,552143	2,18E-05	0,0005161	2,531132
ENSG00000231806	PCAT7	1,178994	-0,037014	3,871721	0,00024	0,0028498	0,419904
ENSG00000103056	SMPD3	1,17648	4,454114	5,265522	1,47E-06	8,46E-05	5,018537
ENSG00000166143	PPP1R14D	1,175246	1,9190075	2,774385	0,007086	0,033252	-2,59642
ENSG00000237289	CKMT1B	1,173879	2,2947615	4,379786	4,08E-05	0,0007974	2,012999
ENSG00000204613	TRIM10	1,173787	1,5165538	4,238435	6,76E-05	0,0011486	1,565877
ENSG00000279017	NA	1,172858	-0,260031	3,401617	0,001111	0,0088431	-0,89301
ENSG00000221947	XKR9	1,172839	2,6609963	3,930864	0,000197	0,0024691	0,555559

ENSG00000154274	C4orf19	1,171233	4,2385978	6,021722	7,18E-08	1,28E-05	7,910312
ENSG00000120306	CYSTM1	1,170892	7,6699309	5,600035	3,92E-07	3,72E-05	6,247905
ENSG00000114455	HHLA2	1,169939	3,9833602	2,827856	0,006107	0,0299094	-2,71797
ENSG00000131781	FMO5	1,16856	4,4325837	4,661758	1,46E-05	0,0003918	2,835628
ENSG00000068615	REEP1	1,166137	2,7910951	3,646245	0,000508	0,0050009	-0,32072
ENSG00000133019	CHRM3	1,165729	2,2641355	3,153178	0,002379	0,0152426	-1,66707
ENSG00000021488	SLC7A9	1,165195	-0,346439	2,630114	0,010489	0,044276	-2,76181
ENSG00000166165	CKB	1,161282	5,206172	4,837272	7,56E-06	0,0002465	3,406655
ENSG00000154930	ACSS1	1,160937	5,5078447	6,711791	4,18E-09	2,35E-06	10,61367
ENSG00000148735	PLEKHS1	1,15935	5,0800199	3,189393	0,002134	0,0141539	-1,88968
ENSG00000164237	CMBL	1,158511	5,376995	6,124247	4,73E-08	1,04E-05	8,272741
ENSG00000196711	ALKAL1	1,157846	0,4394505	2,98541	0,0039	0,0218117	-1,97592
ENSG00000064787	BCAS1	1,156068	5,9219717	3,818407	0,000287	0,0032671	-0,06564
ENSG00000245848	CEBPA	1,151643	2,7908094	3,742554	0,00037	0,0039426	-0,03191
ENSG00000129173	E2F8	1,150613	2,601342	6,660828	5,17E-09	2,66E-06	10,34028
ENSG00000114812	VIPR1	1,147865	3,1389323	4,13474	9,73E-05	0,0014797	1,16874
ENSG00000133710	SPINK5	1,147044	2,7128249	3,231242	0,00188	0,012917	-1,49768
ENSG00000182253	SYNM	1,145693	3,7907114	2,86197	0,005549	0,0279715	-2,60444
ENSG00000160145	KALRN	1,142897	5,0663923	6,034377	6,82E-08	1,25E-05	7,92889
ENSG00000242515	UGT1A10	1,142402	3,3392882	2,613757	0,010957	0,045746	-3,14185
ENSG00000145287	PLAC8	1,138997	5,7023881	3,313381	0,001462	0,0107701	-1,57578
ENSG00000104808	DHDH	1,133083	0,1942601	3,847981	0,00026	0,0030333	0,358414
ENSG00000236699	ARHGEF38	1,131699	3,9722864	4,136481	9,67E-05	0,001472	1,095719
ENSG00000185015	CA13	1,129936	4,288955	6,556862	7,96E-09	3,32E-06	10,01578
ENSG00000255394	C8orf49	1,128354	-1,228544	2,857486	0,00562	0,0282167	-2,2322
ENSG00000120756	PLS1	1,128286	7,3017328	5,436332	7,50E-07	5,61E-05	5,615535
ENSG00000141448	GATA6	1,1252	5,2673062	5,308546	1,24E-06	7,47E-05	5,134806
ENSG00000234155	LINC02535	1,124805	0,2538774	3,125715	0,002583	0,0161707	-1,61691
ENSG00000253368	TRNP1	1,122273	5,7531888	3,149312	0,002407	0,0153606	-2,03658
ENSG00000253598	SLC10A5	1,121225	0,8758658	5,119417	2,58E-06	0,0001244	4,475094
ENSG00000172831	CES2	1,116968	5,7593432	6,086048	5,53E-08	1,11E-05	8,114687
ENSG00000114854	TNNC1	1,116063	2,0204454	2,99495	0,003794	0,0213824	-2,05897
ENSG00000085276	MECOM	1,115646	6,3857199	6,596487	6,75E-09	3,12E-06	10,14444
ENSG00000189221	MAOA	1,115481	5,5532856	4,633006	1,62E-05	0,0004189	2,665436
ENSG00000260337	NA	1,114851	-0,052415	3,08818	0,002887	0,0175233	-1,70176
ENSG00000129007	CALML4	1,107976	2,311824	4,718467	1,18E-05	0,0003365	3,152207
ENSG00000176920	FUT2	1,107008	5,4387442	5,138409	2,40E-06	0,0001181	4,494879
ENSG00000109654	TRIM2	1,105688	6,8361794	7,022441	1,14E-09	1,16E-06	11,86957
ENSG00000279427	NA	1,104034	-0,565695	3,743515	0,000369	0,0039374	0,029551
ENSG00000078399	HOXA9	1,103326	-0,301754	3,173492	0,002239	0,0145995	-1,4832
ENSG00000099812	MISP	1,102595	6,4751181	5,547072	4,84E-07	4,39E-05	6,02033
ENSG00000100079	LGALS2	1,102349	2,6720995	2,594706	0,011525	0,0475135	-3,09757
ENSG00000157111	TMEM171	1,102203	2,4110867	5,033147	3,60E-06	0,0001557	4,248896
ENSG00000109265	KIAA1211	1,09571	4,148173	4,733379	1,12E-05	0,0003255	3,111433

ENSG00000115616	SLC9A2	1,090127	3,5622169	3,038363	0,003343	0,0195622	-2,11685
ENSG00000261616	NA	1,090076	-0,102444	3,716929	0,000403	0,0042192	-0,02606
ENSG00000274750	HIST1H3E	1,089936	1,603432	2,77667	0,007042	0,0330886	-2,56382
ENSG00000179588	ZFPM1	1,088715	3,2583605	5,192142	1,95E-06	0,0001039	4,811795
ENSG00000260912	NA	1,087819	3,5860077	4,004228	0,000153	0,0020499	0,708507
ENSG00000135324	MRAP2	1,087269	2,9145473	3,433587	0,001005	0,0082565	-0,95216
ENSG00000006047	YBX2	1,085573	-0,570106	2,608173	0,011121	0,0461842	-2,79859
ENSG00000105523	FAM83E	1,084459	5,0635122	5,75666	2,10E-07	2,54E-05	6,848667
ENSG00000188761	BCL2L15	1,079672	5,7130885	4,332036	4,84E-05	0,0009061	1,619819
ENSG00000162981	FAM84A	1,079543	4,126277	3,271382	0,001663	0,0117951	-1,55902
ENSG00000135697	BCO1	1,0771	1,7784437	3,827089	0,000279	0,003199	0,281943
ENSG00000224259	LINC01133	1,075615	4,4522723	3,021819	0,003508	0,0201535	-2,28043
ENSG00000250508	LOC105369364	1,074367	-0,478611	4,132927	9,79E-05	0,0014854	1,159568
ENSG00000196167	COLCA1	1,069647	4,4322366	3,132538	0,002531	0,0159127	-1,98075
ENSG00000172828	CES3	1,068844	1,8848871	2,703658	0,008603	0,0383988	-2,76134
ENSG00000139835	GRTP1	1,063406	4,0271776	7,086225	8,72E-10	9,82E-07	12,13099
ENSG00000113494	PRLR	1,062766	2,5844841	2,982636	0,003931	0,021898	-2,14309
ENSG00000235280	NA	1,060712	-0,778946	3,217994	0,001957	0,0132671	-1,37172
ENSG00000139874	SSTR1	1,058823	2,4778243	2,951877	0,004296	0,023346	-2,21079
ENSG00000156966	B3GNT7	1,05868	5,1680499	5,36348	9,99E-07	6,48E-05	5,345241
ENSG00000158467	AHCYL2	1,058586	6,2628116	5,089365	2,90E-06	0,0001329	4,300453
ENSG00000243896	OR2A7	1,057742	-0,887647	3,229771	0,001889	0,012954	-1,34388
ENSG00000262714	NA	1,056749	-0,556845	2,652265	0,009885	0,0424309	-2,70367
ENSG00000119125	GDA	1,05235	5,6742248	4,062324	0,000125	0,0017735	0,722332
ENSG00000170522	ELOVL6	1,050143	4,7155804	5,72303	2,40E-07	2,70E-05	6,734536
ENSG00000152782	PANK1	1,049259	2,5747741	5,885392	1,25E-07	1,80E-05	7,372162
ENSG00000177076	ACER2	1,04919	2,3044378	3,925656	0,0002	0,0025007	0,560056
ENSG00000204991	SPIRE2	1,046372	4,7635823	3,994044	0,000158	0,0021068	0,554734
ENSG00000143850	PLEKHA6	1,046052	6,4900501	5,999571	7,86E-08	1,30E-05	7,772755
ENSG00000105289	TJP3	1,043911	5,5786303	5,067021	3,16E-06	0,0001415	4,227168
ENSG00000280027	NA	1,04249	-0,953324	2,687036	0,009	0,0395786	-2,61404
ENSG00000166582	CENPV	1,041633	4,0233329	5,590761	4,07E-07	3,82E-05	6,263635
ENSG00000145703	IQGAP2	1,040902	6,1118671	4,56184	2,11E-05	0,0005033	2,405126
ENSG00000186564	FOXD2	1,040487	1,363013	4,389487	3,94E-05	0,0007751	2,051579
ENSG00000161267	BDH1	1,036521	4,1380064	6,16187	4,06E-08	9,62E-06	8,459854
ENSG00000244479	NA	1,036394	0,1728057	4,07598	0,000119	0,0017192	1,034111
ENSG00000167617	CDC42EP5	1,03478	4,1543322	5,164751	2,17E-06	0,0001119	4,665774
ENSG00000178882	RFLNA	1,033986	4,8021991	3,642023	0,000515	0,0050592	-0,55104
ENSG00000147041	SYTL5	1,031724	3,2054492	2,603767	0,011251	0,0465881	-3,146
ENSG00000125046	SSUH2	1,030729	-0,758723	3,010276	0,003628	0,0207041	-1,87994
ENSG00000154310	TNIK	1,029445	5,7503687	5,95094	9,58E-08	1,50E-05	7,58459
ENSG00000141655	TNFRSF11A	1,028654	4,7281969	4,269678	6,05E-05	0,0010602	1,464362
ENSG00000229417	NA	1,025868	0,007017	3,933052	0,000195	0,0024573	0,600945
ENSG00000149260	CAPN5	1,023026	7,099976	4,941361	5,10E-06	0,0001948	3,773119

ENSG00000234602	MCIDAS	1,021688	-0,771737	2,867381	0,005465	0,0277262	-2,2148
ENSG00000157613	CREB3L1	1,019746	6,1014858	4,821323	8,03E-06	0,0002578	3,324842
ENSG00000143416	SELENBP1	1,019022	5,6605007	4,716583	1,19E-05	0,0003378	2,957655
ENSG00000177459	ERICH5	1,018102	2,7280147	2,817004	0,006295	0,0305493	-2,57491
ENSG00000101049	SGK2	1,017926	3,3607588	3,853441	0,000255	0,0029877	0,256494
ENSG00000169684	CHRNA5	1,017371	2,2177337	4,221114	7,18E-05	0,0012019	1,497643
ENSG00000178401	DNAJC22	1,013219	4,8465028	4,923105	5,47E-06	0,0002046	3,736791
ENSG00000130751	NPAS1	1,01162	-0,332103	3,614549	0,000563	0,0053544	-0,31855
ENSG00000131187	F12	1,010851	1,8282618	3,954086	0,000182	0,002321	0,667576
ENSG00000162437	RAVER2	1,008461	4,9094751	6,902755	1,88E-09	1,41E-06	11,39399
ENSG00000272405	NA	1,008207	3,1645211	3,403331	0,001105	0,0088074	-1,06477
ENSG00000101447	FAM83D	1,004705	3,7718222	3,228326	0,001897	0,0129664	-1,63211
ENSG00000043591	ADRB1	1,00428	-0,795078	2,76175	0,007338	0,034045	-2,45373
ENSG00000171714	ANO5	1,003079	2,7719594	3,453002	0,000945	0,0078877	-0,88305
ENSG00000164045	CDC25A	1,000101	1,9240816	4,82547	7,91E-06	0,0002553	3,517906

Pancreatic Cluster

Ensmbl_ID	Gene	logFC	AveExpr	t	P.Value	adj.P.Val	B
ENSG00000168928	CTRB2	6,847604	3,6095713	8,365605	3,92E-12	5,57E-09	17,34192
ENSG00000187021	PNLIPRP1	6,835675	1,7847187	8,94107	3,43E-13	1,55E-09	19,69899
ENSG00000142789	CELA3A	6,531977	4,1375728	8,3093	4,97E-12	6,40E-09	17,07212
ENSG00000204787	REG1CP	6,503128	0,1976389	9,333258	6,58E-14	1,00E-09	21,0394
ENSG00000219073	CELA3B	6,443721	1,9724554	8,448548	2,76E-12	4,97E-09	17,69885
ENSG00000168925	CTRB1	6,429738	2,7518822	8,387391	3,57E-12	5,57E-09	17,4593
ENSG00000137392	CLPS	6,398282	2,6098127	8,479567	2,42E-12	4,97E-09	17,83936
ENSG00000153002	CPB1	6,334931	5,7996431	7,803574	4,24E-11	3,54E-08	14,86206
ENSG00000091704	CPA1	6,334445	4,9959499	7,602816	9,91E-11	7,44E-08	14,0581
ENSG00000162438	CTRC	6,327887	3,1263676	8,745589	7,84E-13	2,83E-09	18,93425
ENSG00000143954	REG3G	6,320961	1,2012641	8,240229	6,66E-12	8,00E-09	16,80954
ENSG00000158516	CPA2	6,293267	3,5290388	8,501601	2,20E-12	4,97E-09	17,911
ENSG00000175535	PNLIP	6,26705	4,7652436	7,284429	3,80E-10	2,45E-07	12,75
ENSG00000170890	PLA2G1B	6,100086	2,8026156	8,359631	4,02E-12	5,57E-09	17,34433
ENSG00000266200	PNLIPRP2	6,056769	2,4503076	7,514451	1,44E-10	9,89E-08	13,86888
ENSG00000240338	LOC100506281	6,026825	1,4106728	9,004121	2,63E-13	1,55E-09	19,92463
ENSG00000172023	REG1B	5,972429	3,6486544	7,170331	6,15E-10	3,82E-07	12,38276
ENSG00000215704	CELA2B	5,8002	0,2189002	7,764455	5,00E-11	3,92E-08	14,79576
ENSG00000204983	PRSS1	5,729825	5,8697781	7,799104	4,32E-11	3,54E-08	14,84201
ENSG00000179751	SYCN	5,700911	0,0274263	7,507815	1,48E-10	9,89E-08	13,7484
ENSG00000142615	CELA2A	5,652016	1,3061529	6,641476	5,62E-09	2,41E-06	10,34927
ENSG00000124232	RBPJL	5,569718	-0,603487	8,666697	1,09E-12	3,29E-09	18,21254
ENSG00000172016	REG3A	5,526682	4,6953402	6,547126	8,32E-09	3,26E-06	9,722329
ENSG00000170835	CEL	5,456262	4,2028641	7,973049	2,07E-11	2,07E-08	15,66438
ENSG00000114204	SERPINI2	5,414759	-0,050241	8,073022	1,35E-11	1,52E-08	15,98835
ENSG00000243480	AMY2A	5,305541	0,4014104	6,125297	4,72E-08	1,18E-05	8,319984
ENSG00000275896	PRSS2	4,980066	6,3159814	6,900385	1,91E-09	9,81E-07	11,10078
ENSG00000103375	AQP8	4,871254	-1,141602	7,910169	2,70E-11	2,56E-08	15,11752
ENSG00000169347	GP2	4,849135	4,6690387	6,148946	4,29E-08	1,12E-05	8,116693
ENSG00000240521	NA	4,801809	-1,756758	7,986673	1,95E-11	2,07E-08	15,23076
ENSG00000254647	INS	4,624809	5,2157832	4,876338	6,54E-06	0,0005427	3,167986
ENSG00000115263	GCG	4,474246	5,6478546	4,144457	9,41E-05	0,0045967	0,572762
ENSG00000118271	TTR	4,43078	4,906332	4,82631	7,89E-06	0,0006237	3,011463
ENSG00000164756	SLC30A8	4,34726	2,9540053	5,558111	4,64E-07	6,80E-05	5,999009
ENSG00000108849	PPY	4,159823	3,3614227	4,399347	3,81E-05	0,0021773	1,706475
ENSG00000213373	LINC00671	3,994046	-1,13059	9,208636	1,11E-13	1,00E-09	20,13656
ENSG00000006071	ABCC8	3,954627	2,8982318	5,86327	1,37E-07	2,56E-05	7,18708
ENSG00000138161	CUZD1	3,872991	3,3698715	7,54543	1,26E-10	9,11E-08	13,95406
ENSG00000115386	REG1A	3,76818	7,8704594	5,460198	6,84E-07	9,27E-05	5,384563
ENSG00000095981	KCNK16	3,764459	0,3057747	5,775812	1,95E-07	3,47E-05	6,979209
ENSG00000121351	IAPP	3,753284	-0,013798	5,249273	1,56E-06	0,0001795	5,020207
ENSG00000089199	CHGB	3,73845	4,5283488	5,305812	1,25E-06	0,0001497	4,836319
ENSG00000106927	AMBP	3,707702	2,3515404	7,097003	8,37E-10	4,86E-07	12,16478

ENSG00000152254	G6PC2	3,695383	0,8901466	5,306834	1,25E-06	0,0001497	5,200225
ENSG00000136750	GAD2	3,687659	0,6122514	6,194287	3,56E-08	9,72E-06	8,588809
ENSG00000149654	CDH22	3,655351	-0,738222	7,849562	3,49E-11	3,14E-08	14,97488
ENSG00000125851	PCSK2	3,591869	2,0159907	5,476361	6,42E-07	8,97E-05	5,769193
ENSG00000145642	SHISAL2B	3,452455	-0,573324	6,90876	1,84E-09	9,81E-07	11,31291
ENSG00000162992	NEUROD1	3,426828	0,754405	5,462887	6,77E-07	9,24E-05	5,787963
ENSG00000079689	SCGN	3,308419	2,1492762	5,86256	1,37E-07	2,56E-05	7,243785
ENSG00000132693	CRP	3,266568	4,3898595	4,043018	0,000134	0,0060791	0,350316
ENSG00000104760	FGL1	3,242747	0,2172018	7,098328	8,32E-10	4,86E-07	12,13759
ENSG00000278041	NA	3,23659	-0,426718	6,195947	3,54E-08	9,72E-06	8,564854
ENSG00000126266	FFAR1	3,208746	-0,573258	6,709371	4,24E-09	1,86E-06	10,53605
ENSG00000188338	SLC38A3	3,194633	0,1846092	6,365024	1,77E-08	5,65E-06	9,246689
ENSG00000124701	APOBEC2	3,185473	0,5928473	8,470872	2,51E-12	4,97E-09	17,67886
ENSG00000137731	FXYD2	3,180994	2,1170946	6,716255	4,12E-09	1,86E-06	10,63047
ENSG00000176840	MIR7-3HG	3,180656	-0,123357	5,737026	2,27E-07	3,87E-05	6,830785
ENSG00000100604	CHGA	3,171912	3,5196661	4,185074	8,16E-05	0,0041089	0,954256
ENSG00000171759	PAH	3,164389	0,3791652	5,91914	1,09E-07	2,24E-05	7,525807
ENSG00000120057	SFRP5	3,159178	2,4484143	5,738718	2,26E-07	3,87E-05	6,741717
ENSG00000185559	DLK1	3,147377	0,1579883	4,825052	7,93E-06	0,000624	3,491684
ENSG00000153822	KCNJ16	3,103179	2,264971	5,067047	3,16E-06	0,0003096	4,219453
ENSG00000185615	PDIA2	3,074729	2,666831	5,628485	3,51E-07	5,36E-05	6,297674
ENSG00000104112	SCG3	3,074284	1,946095	5,887658	1,24E-07	2,40E-05	7,353822
ENSG00000159409	CELF3	3,068427	0,6399183	5,972354	8,80E-08	1,89E-05	7,728649
ENSG00000182759	MAFA	3,037852	-1,259719	6,379214	1,67E-08	5,65E-06	9,187597
ENSG00000125820	NKX2-2	3,030193	-0,775167	6,104261	5,15E-08	1,27E-05	8,192139
ENSG00000157005	SST	3,023719	3,0485778	4,175428	8,44E-05	0,0042269	0,991896
ENSG00000163499	CRYBA2	3,011529	-0,225776	4,892834	6,14E-06	0,0005171	3,744956
ENSG00000231013	LOC107105282	3,008623	2,480069	6,759639	3,44E-09	1,63E-06	10,78976
ENSG00000158014	SLC30A2	2,995848	0,939618	6,475083	1,12E-08	4,04E-06	9,690532
ENSG00000185002	RFX6	2,987055	0,0011933	4,987072	4,29E-06	0,0003849	4,072746
ENSG00000256969	NA	2,976776	-2,149111	6,22863	3,09E-08	8,99E-06	8,484654
ENSG00000170827	CELP	2,953042	-1,851274	4,816637	8,18E-06	0,0006384	3,472786
ENSG00000126259	KIRREL2	2,942676	0,6034419	5,669738	2,98E-07	4,79E-05	6,572233
ENSG00000133169	BEX1	2,916615	1,4383549	6,477621	1,11E-08	4,04E-06	9,694902
ENSG00000145920	CPLX2	2,905437	0,9075674	4,976101	4,48E-06	0,0003974	3,988972
ENSG00000132703	APCS	2,896988	2,563928	3,799126	0,000307	0,0109831	-0,16264
ENSG00000169550	MUC15	2,88657	-0,216147	4,763265	1,00E-05	0,0007537	3,290239
ENSG00000144406	UNC80	2,886062	0,9274175	6,358268	1,82E-08	5,65E-06	9,229917
ENSG00000101746	NOL4	2,884697	-0,604249	5,903834	1,16E-07	2,30E-05	7,444475
ENSG00000173826	KCNH6	2,877538	-0,996941	6,562137	7,81E-09	3,13E-06	9,918995
ENSG00000173452	TMEM196	2,863642	-1,507076	6,767241	3,33E-09	1,62E-06	10,62655
ENSG00000178473	UCN3	2,836276	0,0730503	5,205517	1,85E-06	0,0002036	4,858791
ENSG00000145198	VWA5B2	2,830941	0,8653181	6,630647	5,88E-09	2,46E-06	10,30628
ENSG00000152779	SLC16A12	2,826632	0,7833723	6,907264	1,85E-09	9,81E-07	11,40594

ENSG00000091583	APOH	2,825721	0,1137474	6,20502	3,41E-08	9,64E-06	8,623276
ENSG00000171303	KCNK3	2,814514	1,5511484	6,371222	1,72E-08	5,65E-06	9,269991
ENSG00000130294	KIF1A	2,782882	2,136292	5,664521	3,04E-07	4,82E-05	6,479
ENSG00000186487	MYT1L	2,772592	-1,707972	6,489297	1,06E-08	3,97E-06	9,536754
ENSG00000166111	SVOP	2,768299	-0,502534	6,314518	2,17E-08	6,64E-06	9,015122
ENSG00000159248	GJD2	2,752468	-0,248332	5,077555	3,04E-06	0,0003004	4,402843
ENSG00000170099	SERPINA6	2,731418	1,4236913	5,06521	3,18E-06	0,0003101	4,278683
ENSG00000244067	GSTA2	2,728582	0,0603509	5,13616	2,42E-06	0,0002524	4,607216
ENSG00000120251	GRIA2	2,701885	0,0023134	6,159542	4,11E-08	1,09E-05	8,444326
ENSG00000161270	NPHS1	2,694181	-0,413413	5,344921	1,08E-06	0,000132	5,372353
ENSG00000088926	F11	2,689181	0,6335599	5,424444	7,88E-07	0,0001007	5,648852
ENSG00000107295	SH3GL2	2,687902	-0,353984	5,903481	1,16E-07	2,30E-05	7,453823
ENSG00000236780	LINC01829	2,682382	-0,729908	6,20401	3,42E-08	9,64E-06	8,574973
ENSG00000132872	SYT4	2,650788	1,0235966	5,11355	2,64E-06	0,0002677	4,481036
ENSG00000171557	FGG	2,644639	2,5929959	3,289463	0,001574	0,0359993	-1,6922
ENSG00000177511	ST8SIA3	2,620759	2,1139066	5,81393	1,67E-07	3,04E-05	7,057277
ENSG00000178171	AMER3	2,611768	-1,372915	5,9837	8,41E-08	1,85E-05	7,685928
ENSG00000228262	LINC01317	2,595548	-0,951523	5,329603	1,14E-06	0,0001392	5,307237
ENSG00000171560	FGA	2,580972	2,6247197	3,648931	0,000504	0,0157577	-0,63579
ENSG00000163631	ALB	2,577195	1,1927915	4,85089	7,19E-06	0,000584	3,52011
ENSG00000151952	TMEM132D	2,552129	-1,146213	5,908054	1,14E-07	2,30E-05	7,424719
ENSG00000130643	CALY	2,54243	-0,674462	5,922779	1,08E-07	2,23E-05	7,512225
ENSG00000189292	ALKAL2	2,534332	0,4401773	6,894111	1,96E-09	9,81E-07	11,34018
ENSG00000167434	CA4	2,532992	-0,48548	5,451069	7,09E-07	9,53E-05	5,760881
ENSG00000163623	NKX6-1	2,531826	0,446224	6,598238	6,73E-09	2,76E-06	10,16942
ENSG00000139055	ERP27	2,52586	3,0816363	6,053708	6,32E-08	1,52E-05	7,918123
ENSG00000100314	CABP7	2,519797	0,8009805	6,041389	6,65E-08	1,56E-05	7,993389
ENSG00000250366	TUNAR	2,51597	-0,296402	5,650155	3,22E-07	4,96E-05	6,502171
ENSG00000268555	NA	2,515286	-1,546324	6,032247	6,90E-08	1,57E-05	7,847478
ENSG00000136698	CFC1	2,510183	-2,17805	5,169141	2,13E-06	0,0002248	4,666298
ENSG00000181617	FDCSP	2,50296	0,1343237	3,91734	0,000206	0,0083787	0,459264
ENSG00000243709	LEFTY1	2,500343	1,5633682	4,170572	8,59E-05	0,0042641	1,144984
ENSG00000183166	CALN1	2,499719	-1,822937	5,288145	1,34E-06	0,0001593	5,113211
ENSG00000186369	LINC00643	2,483817	0,3395045	5,124934	2,53E-06	0,0002591	4,557234
ENSG00000175262	C1orf127	2,461334	0,6882973	6,391888	1,58E-08	5,58E-06	9,361992
ENSG00000145321	GC	2,447406	3,5454954	3,858682	0,000251	0,0097279	-0,11714
ENSG00000112246	SIM1	2,441336	-0,771287	3,53957	0,000718	0,0204877	-0,61149
ENSG00000054356	PTPRN	2,403422	3,1282003	4,784514	9,23E-06	0,000711	3,096312
ENSG00000102109	PCSK1N	2,383711	2,3205541	5,117426	2,60E-06	0,0002652	4,400079
ENSG00000235142	LINC02532	2,383205	2,7873441	4,478101	2,86E-05	0,0017661	2,056598
ENSG00000138079	SLC3A1	2,364915	4,2463593	5,039137	3,52E-06	0,0003312	3,870351
ENSG00000166828	SCNN1G	2,347449	-1,395375	4,013934	0,000148	0,006555	0,850472
ENSG00000039537	C6	2,347159	3,4547963	5,038508	3,53E-06	0,0003312	3,97863
ENSG00000280119	LOC285097	2,339292	-1,331493	5,733698	2,30E-07	3,88E-05	6,762915

ENSG00000255595	LOC101927531	2,334111	-1,416638	4,403564	3,75E-05	0,0021512	2,097632
ENSG00000175426	PCSK1	2,332974	2,1170797	4,969745	4,59E-06	0,0004032	3,876683
ENSG00000012223	LTF	2,330309	3,10521	4,666068	1,44E-05	0,0010144	2,675905
ENSG00000004848	ARX	2,32431	-0,176849	5,038415	3,53E-06	0,0003312	4,261178
ENSG00000198739	LRRTM3	2,293953	-1,969033	4,657629	1,48E-05	0,0010421	2,932314
ENSG00000169862	CTNND2	2,283232	1,0182371	6,98434	1,34E-09	7,56E-07	11,72
ENSG00000154080	CHST9	2,278149	1,904848	5,530969	5,17E-07	7,40E-05	5,984264
ENSG00000278195	SSTR3	2,274296	-0,465402	5,447588	7,19E-07	9,53E-05	5,748361
ENSG00000080293	SCTR	2,272061	4,0713131	5,31711	1,20E-06	0,0001451	4,93691
ENSG00000167748	KLK1	2,264373	3,2479547	3,869654	0,000242	0,0095239	-0,03539
ENSG00000173404	INSM1	2,260448	-0,063117	4,633104	1,62E-05	0,0011069	2,833035
ENSG00000107954	NEURL1	2,259849	2,0272603	6,359363	1,81E-08	5,65E-06	9,205481
ENSG00000197106	SLC6A17	2,254672	0,4569211	5,619096	3,64E-07	5,52E-05	6,384061
ENSG00000165548	TMEM63C	2,249132	0,4697889	5,589828	4,09E-07	6,10E-05	6,273511
ENSG00000144834	TAGLN3	2,246429	-0,74317	5,691467	2,73E-07	4,51E-05	6,642745
ENSG00000165125	TRPV6	2,222006	3,8220393	4,482697	2,81E-05	0,0017434	1,926056
ENSG00000130226	DPP6	2,217853	0,5396999	5,248296	1,57E-06	0,0001795	5,000035
ENSG00000219159	NA	2,214493	-0,310801	4,089882	0,000114	0,0052608	1,039014
ENSG00000133958	UNC79	2,212866	0,2987619	6,135646	4,53E-08	1,16E-05	8,358878
ENSG00000138798	EGF	2,205623	0,6851231	4,260436	6,26E-05	0,0032767	1,521496
ENSG00000124713	GNMT	2,204701	-0,309342	6,036799	6,77E-08	1,57E-05	7,962224
ENSG00000241935	HOGA1	2,20153	0,4453759	6,04201	6,63E-08	1,56E-05	7,998299
ENSG00000001626	CFTR	2,198806	6,7381233	4,76612	9,89E-06	0,0007489	2,739686
ENSG00000013293	SLC7A14	2,198387	-0,39501	5,001954	4,06E-06	0,0003748	4,135175
ENSG00000196581	AJAP1	2,196819	0,4485335	6,018461	7,30E-08	1,64E-05	7,907482
ENSG00000146039	SLC17A4	2,192735	1,5681437	3,719117	0,0004	0,013449	-0,29691
ENSG00000106633	GCK	2,171808	-0,285886	5,962489	9,16E-08	1,92E-05	7,680154
ENSG00000058404	CAMK2B	2,168749	0,5666779	6,007547	7,63E-08	1,70E-05	7,865031
ENSG00000187889	FYB2	2,156853	-0,4879	5,27824	1,40E-06	0,000164	5,129009
ENSG00000144290	SLC4A10	2,154928	0,1002958	5,144232	2,35E-06	0,0002461	4,635353
ENSG00000148798	INA	2,13745	0,1088278	5,462844	6,77E-07	9,24E-05	5,804945
ENSG00000100095	SEZ6L	2,132369	0,9416508	4,415381	3,59E-05	0,002088	2,020509
ENSG00000105707	HPN	2,120084	2,6658924	4,302649	5,38E-05	0,0028956	1,469769
ENSG00000163395	IGFN1	2,110904	1,2762862	4,900981	5,95E-06	0,0005084	3,693701
ENSG00000163497	FEV	2,105863	-1,512881	4,383644	4,03E-05	0,0022822	2,033031
ENSG00000040731	CDH10	2,102748	-1,140351	5,091558	2,88E-06	0,0002897	4,450159
ENSG00000185352	HS6ST3	2,101032	-0,277766	5,657061	3,13E-07	4,87E-05	6,528308
ENSG00000135447	PPP1R1A	2,082232	0,6119685	4,656537	1,49E-05	0,0010421	2,875159
ENSG00000197584	KCNMB2	2,078368	-0,266095	5,357334	1,03E-06	0,0001266	5,418084
ENSG00000115361	ACADL	2,073759	0,8783715	6,0839	5,59E-08	1,36E-05	8,157186
ENSG00000123838	C4BPA	2,071634	2,6556363	4,035701	0,000137	0,0062033	0,582967
ENSG00000166922	SCG5	2,059718	4,9846458	5,689042	2,76E-07	4,52E-05	6,267491
ENSG00000168065	SLC22A11	2,059324	-0,800289	5,469291	6,60E-07	9,15E-05	5,820493
ENSG00000141837	CACNA1A	2,056075	2,2113089	5,971581	8,83E-08	1,89E-05	7,665063

ENSG00000143171	RXRG	2,051422	-1,553728	4,996161	4,15E-06	0,0003774	4,102461
ENSG00000168843	FSTL5	2,050583	-1,244836	3,840458	0,000267	0,0099843	0,310958
ENSG00000258986	TMEM179	2,048483	-0,736398	4,448131	3,19E-05	0,0019166	2,231772
ENSG00000188488	SERPINA5	2,036593	4,6820538	6,133733	4,56E-08	1,16E-05	8,054521
ENSG00000268416	NA	2,023602	-1,91951	3,844825	0,000263	0,009942	0,347804
ENSG00000196136	SERPINA3	1,995425	3,3181376	4,648947	1,53E-05	0,0010559	2,585914
ENSG00000274373	NA	1,995326	-1,425962	4,845322	7,35E-06	0,0005884	3,583974
ENSG00000118160	SLC8A2	1,994635	-0,318259	4,847583	7,28E-06	0,0005872	3,58822
ENSG00000185818	NAT8L	1,98728	-0,031019	6,374501	1,70E-08	5,65E-06	9,27487
ENSG00000184672	RALYL	1,983487	-1,079461	4,520875	2,45E-05	0,0015704	2,482819
ENSG00000146151	HMGCLL1	1,978909	-0,048105	5,231295	1,68E-06	0,0001888	4,955255
ENSG00000163581	SLC2A2	1,978562	-0,141481	3,472791	0,000888	0,0238481	-0,857
ENSG00000128564	VGF	1,970121	0,6935063	4,278914	5,86E-05	0,003123	1,582227
ENSG00000141433	ADCYAP1	1,961211	1,7709565	4,867336	6,76E-06	0,0005564	3,535431
ENSG00000147724	FAM135B	1,960836	-1,220203	5,211728	1,81E-06	0,0002	4,874794
ENSG00000162374	ELAVL4	1,950383	0,5240184	5,362305	1,01E-06	0,0001251	5,421666
ENSG00000116329	OPRD1	1,950378	-1,255419	4,718369	1,18E-05	0,0008658	3,15116
ENSG00000163492	CCDC141	1,949347	-0,129924	5,859651	1,39E-07	2,56E-05	7,294106
ENSG00000170927	PKHD1	1,926547	4,2367055	4,650471	1,52E-05	0,0010559	2,457396
ENSG00000267259	NA	1,921914	0,114014	5,183122	2,02E-06	0,0002168	4,776213
ENSG00000171450	CDK5R2	1,917908	-0,929628	4,603622	1,81E-05	0,0012247	2,759326
ENSG00000104435	STMN2	1,905888	3,8132185	3,908589	0,000212	0,0086118	-0,00036
ENSG00000101306	MYLK2	1,904205	-0,987009	5,448817	7,16E-07	9,53E-05	5,738978
ENSG00000110436	SLC1A2	1,90228	0,7984383	5,442298	7,34E-07	9,59E-05	5,709102
ENSG00000265179	NA	1,899794	-1,453874	4,649041	1,53E-05	0,0010559	2,914583
ENSG00000060709	RIMBP2	1,898131	1,0361039	4,746722	1,06E-05	0,0007919	3,16135
ENSG00000108309	RUNDC3A	1,895526	1,0581914	5,70248	2,61E-07	4,36E-05	6,682219
ENSG00000179915	NRXN1	1,88383	0,5676836	4,220223	7,21E-05	0,0037132	1,398233
ENSG00000185518	SV2B	1,883156	1,8511381	5,035062	3,57E-06	0,0003337	4,137255
ENSG00000156959	LHFPL4	1,881583	-0,824392	4,565758	2,08E-05	0,0013715	2,629249
ENSG00000177669	MBOAT4	1,879803	-0,718307	5,554644	4,71E-07	6,84E-05	6,136919
ENSG00000165899	OTOGL	1,874803	0,1609931	6,282104	2,48E-08	7,46E-06	8,923402
ENSG00000124780	KCNK17	1,872373	0,5731032	5,742604	2,22E-07	3,87E-05	6,849588
ENSG00000214456	PLIN5	1,868503	1,6542316	6,275265	2,55E-08	7,55E-06	8,887089
ENSG00000185736	ADARB2	1,862496	-0,728824	5,076331	3,05E-06	0,0003004	4,401751
ENSG00000105825	TFPI2	1,859149	3,501885	4,378788	4,10E-05	0,0023152	1,614711
ENSG00000164266	SPINK1	1,856975	8,3123092	4,51312	2,52E-05	0,0015984	1,913132
ENSG00000162877	PM20D1	1,854277	-0,606548	4,988671	4,27E-06	0,0003845	4,090354
ENSG00000050555	LAMC3	1,841247	2,0710269	6,180701	3,77E-08	1,01E-05	8,495359
ENSG00000129990	SYT5	1,837823	-0,23197	4,894712	6,10E-06	0,0005158	3,751758
ENSG00000134873	CLDN10	1,832647	4,848876	3,298616	0,001531	0,0353527	-2,00794
ENSG00000054803	CBLN4	1,829498	-0,41585	4,502443	2,62E-05	0,0016503	2,402703
ENSG00000112175	BMP5	1,826454	0,8312173	4,030861	0,00014	0,0062324	0,757779
ENSG00000225329	LHFPL3-AS2	1,823086	0,8478491	3,634907	0,000527	0,0163814	-0,47803

ENSG00000170561	IRX2	1,821864	-0,142648	4,115345	0,000104	0,0048772	1,109892
ENSG00000106536	POU6F2	1,820469	-0,631273	4,194749	7,89E-05	0,0039825	1,394421
ENSG00000256124	LINC01152	1,815749	-0,55213	3,897412	0,00022	0,0088843	0,447684
ENSG00000174672	BRSK2	1,811851	0,5645268	4,266534	6,12E-05	0,0032187	1,551718
ENSG00000092850	TEKT2	1,809778	-0,606373	4,918421	5,57E-06	0,0004805	3,842464
ENSG00000170091	NSG2	1,799689	-0,616419	4,692529	1,30E-05	0,0009417	3,056858
ENSG00000113296	THBS4	1,799426	5,4704483	4,434879	3,35E-05	0,0020041	1,573852
ENSG00000116183	PAPPA2	1,796265	0,8502442	3,703611	0,000421	0,0138951	-0,27008
ENSG00000188175	HEPACAM2	1,792598	1,203387	3,447865	0,000961	0,025201	-1,07055
ENSG00000106236	NPTX2	1,790776	2,4261429	5,75631	2,11E-07	3,72E-05	6,811577
ENSG00000149972	CNTN5	1,784473	-1,325606	3,85246	0,000256	0,0098343	0,351015
ENSG00000181234	TMEM132C	1,784332	0,0113961	4,562173	2,11E-05	0,0013846	2,58588
ENSG00000124493	GRM4	1,778114	-0,65948	3,654842	0,000494	0,0155373	-0,28125
ENSG00000152092	ASTN1	1,773247	-1,348188	4,28061	5,82E-05	0,0031134	1,696084
ENSG00000109832	DDX25	1,771256	-1,386338	5,445664	7,25E-07	9,53E-05	5,707208
ENSG00000163121	NEURL3	1,770249	2,2943436	5,581809	4,23E-07	6,24E-05	6,150028
ENSG00000206549	NA	1,769681	-0,256682	4,421659	3,51E-05	0,0020679	2,123791
ENSG00000197444	OGDHL	1,769069	1,0106725	5,059955	3,25E-06	0,0003131	4,286348
ENSG00000157423	HYDIN	1,767524	-0,887263	4,449317	3,18E-05	0,0019147	2,24001
ENSG00000107518	ATRNL1	1,764419	0,6356843	4,75275	1,04E-05	0,0007774	3,210379
ENSG00000230461	NA	1,763717	-0,681327	4,847031	7,30E-06	0,0005872	3,593199
ENSG00000108018	SORCS1	1,760248	-0,236247	3,815655	0,00029	0,0104807	0,173182
ENSG00000100448	CTSG	1,757335	-0,298178	3,487032	0,000849	0,0230683	-0,80176
ENSG00000151224	MAT1A	1,756807	-0,007921	3,896698	0,000221	0,0088859	0,406265
ENSG00000164199	ADGRV1	1,755836	0,7100076	5,277417	1,40E-06	0,000164	5,099964
ENSG00000080493	SLC4A4	1,74927	6,5337037	4,100032	0,00011	0,0051178	0,416187
ENSG00000178568	ERBB4	1,748876	-0,770533	5,435883	7,53E-07	9,69E-05	5,699103
ENSG00000166405	RIC3	1,74628	2,382753	4,642433	1,57E-05	0,0010775	2,677415
ENSG00000108576	SLC6A4	1,741026	-0,321726	5,198885	1,90E-06	0,0002077	4,841
ENSG00000164638	SLC29A4	1,740443	2,3969699	5,362051	1,01E-06	0,0001251	5,306531
ENSG00000083067	TRPM3	1,739952	-0,945886	4,9606	4,75E-06	0,0004134	3,991442
ENSG00000242173	ARHGDIG	1,734737	-0,487055	3,567805	0,000655	0,0191083	-0,55094
ENSG00000115290	GRB14	1,725888	2,128647	5,43859	7,45E-07	9,66E-05	5,617746
ENSG00000177103	DSCAML1	1,71104	0,1610012	4,537934	2,30E-05	0,0014924	2,494667
ENSG00000171951	SCG2	1,709121	4,4669296	4,432285	3,38E-05	0,0020041	1,658316
ENSG00000145451	GLRA3	1,707728	-1,435079	4,331802	4,85E-05	0,0026731	1,863397
ENSG00000063015	SEZ6	1,705079	-1,26368	5,247646	1,57E-06	0,0001795	5,001395
ENSG00000157542	KCNJ6	1,70012	0,6389215	4,078376	0,000118	0,0054473	0,928713
ENSG00000147488	ST18	1,69985	0,9710685	4,87668	6,53E-06	0,0005427	3,627747
ENSG00000145087	STXBP5L	1,698633	-1,118616	3,754119	0,000356	0,0122748	0,043967
ENSG00000015413	DPEP1	1,691161	3,9720923	3,683991	0,000449	0,0144762	-0,73492
ENSG00000152578	GRIA4	1,687315	0,2991887	5,599347	3,94E-07	5,92E-05	6,312451
ENSG00000240038	AMY2B	1,674266	4,537903	4,45804	3,08E-05	0,0018613	1,73902
ENSG00000117707	PROX1	1,673476	3,4035423	6,357898	1,82E-08	5,65E-06	9,097697

ENSG00000160716	CHRNB2	1,667966	-0,917332	4,969682	4,59E-06	0,0004032	4,023529
ENSG00000101349	PAK5	1,663633	-1,440021	3,927596	0,000199	0,0082028	0,585009
ENSG00000170743	SYT9	1,662802	-1,037797	4,459715	3,06E-05	0,0018563	2,278003
ENSG00000077264	PAK3	1,662192	2,9007438	5,899351	1,18E-07	2,32E-05	7,328045
ENSG00000134962	KLB	1,655094	-0,995778	4,678846	1,37E-05	0,0009829	3,015896
ENSG00000163075	CFAP221	1,654819	2,8325478	4,600751	1,83E-05	0,0012285	2,479664
ENSG00000150551	LYPD1	1,653617	1,3849716	5,262593	1,48E-06	0,0001726	5,00835
ENSG00000258793	NA	1,651199	-1,260833	4,580073	1,97E-05	0,0013061	2,682974
ENSG00000215644	GCGR	1,646942	-0,948101	3,663943	0,00048	0,0151361	-0,23484
ENSG00000148702	HABP2	1,644128	3,0096209	3,308891	0,001483	0,0345233	-1,69705
ENSG00000157502	MUM1L1	1,642692	1,9889134	4,757732	1,02E-05	0,0007663	3,124468
ENSG00000178162	FAR2P2	1,637783	-0,945882	5,243668	1,60E-06	0,0001811	4,997466
ENSG00000138356	AOX1	1,633192	3,0887581	5,182277	2,03E-06	0,0002168	4,560204
ENSG00000066032	CTNNA2	1,629607	-0,813528	4,102158	0,000109	0,0050932	1,105676
ENSG00000163873	GRIK3	1,628733	0,2439029	3,946824	0,000186	0,0078261	0,542835
ENSG00000132938	MTUS2	1,627177	0,0727521	5,0504	3,37E-06	0,0003213	4,297389
ENSG00000053108	FSTL4	1,626776	-0,590491	3,590246	0,00061	0,0181083	-0,47708
ENSG00000105290	APLP1	1,622524	2,6529977	4,992419	4,21E-06	0,0003809	3,907397
ENSG00000109072	VTN	1,612847	0,6928405	5,859279	1,39E-07	2,56E-05	7,293002
ENSG00000144583	MARCH4	1,609735	-0,408393	3,955841	0,000181	0,0076068	0,620494
ENSG00000099866	MADCAM1	1,604475	-0,388415	3,695371	0,000433	0,0141732	-0,1804
ENSG00000148053	NTRK2	1,598238	3,7215892	5,060744	3,24E-06	0,0003131	4,023553
ENSG00000103154	NECAB2	1,584409	-0,100736	4,827907	7,84E-06	0,0006227	3,512345
ENSG00000182836	PLCXD3	1,559661	2,1912091	4,066712	0,000123	0,0056433	0,739417
ENSG00000065609	SNAP91	1,557705	0,7212135	4,433716	3,36E-05	0,0020041	2,100618
ENSG00000152932	RAB3C	1,554381	1,6557177	4,500255	2,64E-05	0,0016573	2,250844
ENSG00000164344	KLKB1	1,553555	0,8017891	5,539647	5,00E-07	7,20E-05	6,074492
ENSG00000007372	PAX6	1,548353	3,3397621	5,659853	3,10E-07	4,85E-05	6,347656
ENSG00000155657	TTN	1,542876	-0,475552	5,77955	1,92E-07	3,46E-05	6,982259
ENSG00000104059	FAM189A1	1,541391	0,4744465	4,504156	2,60E-05	0,0016458	2,358993
ENSG00000178821	TMEM52	1,540697	0,0022351	4,346726	4,60E-05	0,0025654	1,858938
ENSG00000055955	ITIH4	1,536551	-0,562433	5,031176	3,63E-06	0,0003369	4,240872
ENSG00000184905	TCEAL2	1,535705	-0,254228	4,12173	0,000102	0,0048199	1,137733
ENSG00000273259	NA	1,534968	-0,994572	4,23089	6,95E-05	0,0035964	1,526113
ENSG00000144847	IGSF11	1,531709	0,7111554	4,871913	6,65E-06	0,0005493	3,627636
ENSG00000112936	C7	1,529968	7,1977928	3,38779	0,001161	0,0290301	-1,79445
ENSG00000133878	DUSP26	1,526908	0,7276652	4,737501	1,10E-05	0,0008162	3,150642
ENSG00000249715	FER1L5	1,516161	-0,962657	4,904667	5,87E-06	0,0005038	3,795509
ENSG00000198732	SMOC1	1,512197	2,1260583	3,649127	0,000503	0,0157577	-0,57288
ENSG00000176406	RIMS2	1,511877	0,6439392	3,672829	0,000466	0,0148041	-0,34231
ENSG00000146038	DCDC2	1,505834	3,3450916	3,388908	0,001157	0,0290259	-1,51917
ENSG00000155816	FMN2	1,505502	0,1085339	3,446169	0,000966	0,0252673	-0,95774
ENSG00000206579	XKR4	1,504904	-1,026944	3,8168	0,000289	0,0104616	0,22824
ENSG00000260230	FRRS1L	1,50064	0,3734794	3,387537	0,001162	0,0290301	-1,15322

ENSG00000180535	BHLHA15	1,497318	-1,249744	3,572045	0,000646	0,0189101	-0,48556
ENSG00000273108	NA	1,495008	-0,470459	4,309875	5,25E-05	0,0028474	1,762707
ENSG00000058335	RASGRF1	1,494885	0,7803635	4,432139	3,38E-05	0,0020041	2,090568
ENSG00000120457	KCNJ5	1,493799	2,2641563	4,313797	5,17E-05	0,0028163	1,553702
ENSG00000170775	GPR37	1,493455	0,0698381	3,490634	0,000839	0,0229438	-0,82531
ENSG00000171766	GATM	1,49134	7,1153278	4,774221	9,60E-06	0,0007358	2,782845
ENSG00000100626	GALNT16	1,489187	1,8461517	4,769661	9,76E-06	0,0007448	3,179513
ENSG00000135406	PRPH	1,48298	-0,406761	3,710134	0,000412	0,0137763	-0,13462
ENSG00000163618	CADPS	1,482551	3,8401257	4,18152	8,27E-05	0,0041489	0,89235
ENSG00000164825	DEFB1	1,481087	4,5669816	3,732759	0,000382	0,0129531	-0,66998
ENSG00000007174	DNAH9	1,478053	-0,56489	4,829822	7,79E-06	0,000621	3,531462
ENSG00000185070	FLRT2	1,47767	5,0391724	5,739399	2,25E-07	3,87E-05	6,460089
ENSG00000215915	ATAD3C	1,475013	1,6142752	4,726524	1,15E-05	0,0008434	3,045627
ENSG00000171551	ECEL1	1,471581	0,5682648	3,385429	0,001169	0,0291021	-1,18037
ENSG00000156219	ART3	1,464916	-1,26375	3,857591	0,000252	0,0097279	0,363925
ENSG00000125675	GRIA3	1,455927	1,3397706	5,385753	9,17E-07	0,0001156	5,470442
ENSG00000122025	FLT3	1,454745	0,820699	5,406091	8,47E-07	0,0001074	5,572824
ENSG00000164935	DCSTAMP	1,453102	0,1789844	3,767159	0,000341	0,0118921	-0,01017
ENSG00000086619	ERO1B	1,450213	5,2913706	6,709506	4,23E-09	1,86E-06	10,34086
ENSG00000205502	C2CD4B	1,447794	1,4014455	4,469425	2,95E-05	0,0018103	2,166578
ENSG00000167964	RAB26	1,445409	2,4564309	5,524585	5,30E-07	7,53E-05	5,918026
ENSG00000182132	KCNIP1	1,445277	-0,025594	4,31618	5,13E-05	0,0028009	1,759115
ENSG00000149294	NCAM1	1,444732	2,1968965	4,820811	8,06E-06	0,0006312	3,330836
ENSG00000165300	SLITRK5	1,441206	1,5357908	4,009119	0,000151	0,0066319	0,620393
ENSG00000105696	TMEM59L	1,43098	0,6318174	3,841069	0,000267	0,0099843	0,175671
ENSG00000237940	LINC01238	1,430194	0,2332173	4,076946	0,000119	0,0054605	0,958559
ENSG00000267385	NA	1,430147	-1,034521	3,752996	0,000358	0,0122748	0,035945
ENSG00000124939	SCGB2A1	1,430143	1,1759261	3,176568	0,002219	0,0457818	-1,83519
ENSG00000162552	WNT4	1,421826	2,8948501	4,858835	6,98E-06	0,0005719	3,393763
ENSG00000144191	CNGA3	1,421393	-0,948735	3,357832	0,001274	0,0310385	-1,11529
ENSG00000136546	SCN7A	1,407551	3,3618829	3,378188	0,001196	0,0295636	-1,55312
ENSG00000131094	C1QL1	1,405589	-0,460874	3,737325	0,000377	0,0128387	-0,04859
ENSG00000134917	ADAMTS8	1,401558	1,1451135	4,273174	5,98E-05	0,0031703	1,523957
ENSG00000203867	RBM20	1,400331	-0,133142	3,504921	0,000802	0,0221243	-0,76469
ENSG00000165443	PHYHIPL	1,398028	0,9363163	4,030736	0,00014	0,0062324	0,747477
ENSG00000167614	TTYH1	1,39629	0,9786446	4,735739	1,11E-05	0,0008182	3,126785
ENSG00000112164	GLP1R	1,390686	0,4453214	3,173735	0,002238	0,0459288	-1,75778
ENSG00000130988	RGN	1,39055	2,1235669	6,534927	8,75E-09	3,35E-06	9,901886
ENSG00000108551	RASD1	1,389713	2,9421868	4,345829	4,61E-05	0,0025657	1,58167
ENSG00000215217	C5orf49	1,378684	-0,442152	4,029503	0,00014	0,0062435	0,854808
ENSG00000157551	KCNJ15	1,373333	4,9701631	3,7706	0,000337	0,0118025	-0,59689
ENSG00000183775	KCTD16	1,368125	1,228392	3,994158	0,000158	0,0068893	0,601928
ENSG00000258867	LINC01146	1,364152	-0,011557	4,404787	3,73E-05	0,0021486	2,053902
ENSG00000105697	HAMP	1,362013	0,7053209	3,76657	0,000342	0,0118924	-0,06236

ENSG00000197977	ELOVL2	1,361071	0,5030121	4,255143	6,37E-05	0,0033291	1,518922
ENSG00000100867	DHRS2	1,359726	1,4490446	3,487923	0,000846	0,023056	-0,97997
ENSG00000136883	KIF12	1,352414	4,9982019	3,991899	0,00016	0,0069176	0,111443
ENSG00000005102	MEOX1	1,349482	2,6249667	4,097437	0,000111	0,0051509	0,78914
ENSG00000169031	COL4A3	1,34409	2,1572275	4,424627	3,47E-05	0,0020525	1,942836
ENSG00000118785	SPP1	1,339622	9,496688	3,67428	0,000464	0,0148041	-0,79015
ENSG00000271959	NA	1,331287	-0,028994	4,006403	0,000152	0,0066616	0,75332
ENSG00000007402	CACNA2D2	1,330674	2,1359287	5,68029	2,85E-07	4,63E-05	6,539656
ENSG00000116254	CHD5	1,328355	0,1214219	3,456766	0,000934	0,0246991	-0,92846
ENSG00000225649	LOC100506474	1,328249	-0,712002	3,75369	0,000357	0,0122748	0,018317
ENSG00000164946	FREM1	1,326846	2,7919137	3,558289	0,000676	0,0195135	-0,93337
ENSG00000228956	NA	1,324971	0,263342	4,131827	9,84E-05	0,0046899	1,133595
ENSG00000114757	PEX5L	1,32442	-0,987011	3,673724	0,000464	0,0148041	-0,20343
ENSG00000149970	CNKSR2	1,323934	1,670823	5,186959	1,99E-06	0,0002162	4,709326
ENSG00000157315	TMED6	1,317856	0,385318	3,604364	0,000582	0,0176231	-0,5217
ENSG00000116833	NR5A2	1,317082	4,0835035	3,968894	0,000173	0,0073783	0,152311
ENSG00000146530	VWDE	1,316896	1,5921806	3,282477	0,001608	0,0366342	-1,58781
ENSG00000183092	BEGAIN	1,314727	0,8663627	3,956909	0,00018	0,0075968	0,51778
ENSG00000168243	GNG4	1,313204	1,7998038	3,177349	0,002214	0,0457818	-1,90503
ENSG00000099769	IGFALS	1,309239	-0,981965	3,535404	0,000727	0,0207034	-0,60864
ENSG00000148408	CACNA1B	1,307881	-1,030551	3,35714	0,001277	0,0310562	-1,11103
ENSG00000110076	NRXN2	1,306749	2,8326592	4,384142	4,02E-05	0,0022822	1,726908
ENSG00000118004	COLEC11	1,305824	3,6012248	3,60405	0,000583	0,0176231	-0,91995
ENSG00000165495	PKNOX2	1,302818	1,0596419	4,515798	2,49E-05	0,0015885	2,354236
ENSG00000178222	RNF212	1,296986	0,8594725	4,413951	3,61E-05	0,0020921	2,022411
ENSG00000095713	CRTAC1	1,292146	0,4936225	3,662367	0,000482	0,0151861	-0,35866
ENSG00000186715	MST1L	1,290565	1,2670102	3,468804	0,000899	0,0240085	-1,01636
ENSG00000137261	KIAA0319	1,28796	1,4598466	4,689013	1,32E-05	0,0009503	2,925546
ENSG00000130037	KCNA5	1,282793	-0,282974	3,684968	0,000448	0,0144762	-0,21994
ENSG00000204950	LRRC10B	1,27893	-0,101522	4,082251	0,000117	0,0053883	1,00067
ENSG00000007516	BAIAP3	1,27824	3,3812454	4,592343	1,88E-05	0,0012577	2,376603
ENSG00000138650	PCDH10	1,276785	0,001501	3,310784	0,001474	0,0343667	-1,33136
ENSG00000139970	RTN1	1,273166	2,5560232	4,352711	4,50E-05	0,0025188	1,65305
ENSG00000240563	L1TD1	1,268235	-0,943242	3,363863	0,001251	0,0306929	-1,09888
ENSG00000132639	SNAP25	1,267207	2,7180519	4,589325	1,91E-05	0,0012671	2,453203
ENSG00000116147	TNR	1,266505	-1,071345	3,838872	0,000268	0,0099962	0,297699
ENSG00000259663	LOC642366	1,260508	1,5288442	4,144995	9,40E-05	0,0045967	1,063882
ENSG00000170011	MYRIP	1,260426	0,5234799	3,790651	0,000316	0,0112143	0,029826
ENSG00000003989	SLC7A2	1,250501	5,057411	5,862281	1,37E-07	2,56E-05	6,942087
ENSG00000187122	SLIT1	1,249096	0,1044475	4,815221	8,23E-06	0,0006391	3,459485
ENSG00000236279	CLEC2L	1,245242	-1,106457	3,668972	0,000472	0,014966	-0,21032
ENSG00000136842	TMOD1	1,23853	2,1056147	3,974121	0,00017	0,0073169	0,448516
ENSG00000048540	LMO3	1,238052	2,2729859	3,834582	0,000272	0,0100995	-0,01532
ENSG00000160963	COL26A1	1,23762	-0,800091	3,715787	0,000404	0,013547	-0,08991

ENSG00000164061	BSN	1,236959	0,7517876	5,519914	5,40E-07	7,61E-05	6,002078
ENSG00000102003	SYP	1,23547	1,9213581	5,223007	1,73E-06	0,0001926	4,824768
ENSG00000187486	KCNJ11	1,22786	1,1558876	4,408154	3,69E-05	0,0021295	1,977994
ENSG00000011347	SYT7	1,225724	3,6646883	3,523427	0,000756	0,021378	-1,17418
ENSG00000166257	SCN3B	1,225451	0,9321365	4,547453	2,22E-05	0,001456	2,473476
ENSG00000188803	SHISA6	1,214464	0,8404397	3,526703	0,000748	0,0212216	-0,79977
ENSG00000171798	KNDC1	1,208929	1,205068	3,327803	0,001399	0,0329477	-1,41612
ENSG00000187045	TMPRSS6	1,207681	0,4190242	3,872124	0,00024	0,0094655	0,292712
ENSG00000168280	KIF5C	1,205044	2,1811148	4,940143	5,13E-06	0,0004447	3,763265
ENSG00000225792	LOC105375304	1,204186	-0,625097	4,899792	5,98E-06	0,0005084	3,777211
ENSG00000120498	TEX11	1,203387	0,1817772	4,12083	0,000102	0,0048224	1,10427
ENSG00000114279	FGF12	1,20217	0,5298503	4,611604	1,76E-05	0,0011937	2,72472
ENSG00000146215	CRIP3	1,201566	-0,690765	4,343077	4,66E-05	0,0025832	1,881599
ENSG00000101489	CELF4	1,196601	-0,394489	4,536761	2,31E-05	0,0014924	2,517983
ENSG00000118507	AKAP7	1,1949	3,6967787	5,181631	2,03E-06	0,0002168	4,477609
ENSG00000130413	STK33	1,190892	2,0682383	3,935106	0,000194	0,0080697	0,327409
ENSG00000070182	SPTB	1,189282	0,7922463	4,139975	9,56E-05	0,0046069	1,115269
ENSG00000160460	SPTBN4	1,177952	0,8614485	4,115452	0,000104	0,0048772	1,028948
ENSG00000134463	ECHDC3	1,174628	1,617277	4,466625	2,98E-05	0,0018226	2,138379
ENSG00000196109	ZNF676	1,172015	0,3032569	3,858422	0,000251	0,0097279	0,260486
ENSG00000077522	ACTN2	1,169995	-0,349247	3,544939	0,000705	0,0202394	-0,62911
ENSG00000163485	ADORA1	1,167499	2,6213123	4,999032	4,10E-06	0,0003771	3,93496
ENSG00000235098	ANKRD65	1,161366	2,8647802	4,715305	1,20E-05	0,0008722	2,881629
ENSG00000256802	LOC100130111	1,155426	1,0045948	3,964733	0,000175	0,0074312	0,529351
ENSG00000102385	DRP2	1,154258	1,9349153	3,665366	0,000477	0,0151171	-0,50131
ENSG00000183114	FAM43B	1,152527	-0,253465	3,427062	0,001026	0,0263218	-0,97808
ENSG00000196169	KIF19	1,151686	-0,350557	4,031168	0,00014	0,0062324	0,854335
ENSG00000159708	LRRC36	1,15082	1,1767565	4,640423	1,58E-05	0,0010815	2,77726
ENSG00000169432	SCN9A	1,1427	3,3535716	4,272198	6,00E-05	0,0031703	1,273131
ENSG00000223403	MEG9	1,140828	0,1730705	3,31581	0,001452	0,0339697	-1,33498
ENSG00000078018	MAP2	1,137938	3,9684416	5,132159	2,46E-06	0,0002549	4,254556
ENSG00000173947	PIFO	1,137768	1,5395742	3,581411	0,000627	0,0184971	-0,71207
ENSG00000179772	FOXS1	1,133062	2,7080856	4,141843	9,50E-05	0,0045991	0,925424
ENSG00000174871	CNIH2	1,133055	0,2077198	5,129781	2,48E-06	0,0002558	4,579615
ENSG00000130477	UNC13A	1,129295	1,821881	3,29826	0,001532	0,0353527	-1,56961
ENSG00000231789	PIK3CD-AS2	1,129082	-0,548467	3,776263	0,000331	0,0116377	0,075639
ENSG00000130005	GAMT	1,121662	2,7968375	4,98092	4,39E-06	0,0003921	3,849591
ENSG00000103942	HOMER2	1,120077	4,8216233	3,845836	0,000262	0,009942	-0,34277
ENSG00000180616	SSTR2	1,117091	1,8064294	5,663871	3,05E-07	4,82E-05	6,497948
ENSG00000240583	AQP1	1,113174	6,6306488	4,253027	6,42E-05	0,0033445	0,933275
ENSG00000185924	RTN4RL1	1,111848	0,7484211	3,452439	0,000947	0,0249161	-1,00709
ENSG00000123243	ITIH5	1,097599	4,6449246	4,655644	1,49E-05	0,0010421	2,42377
ENSG00000164929	BAALC	1,095181	1,0151504	4,057308	0,000127	0,0058007	0,825821
ENSG00000137868	STRA6	1,093639	4,4880911	3,352068	0,001297	0,0312369	-1,80883

ENSG00000141431	ASXL3	1,092345	1,2959168	3,796913	0,000309	0,0110258	-0,02836
ENSG00000147003	CLTRN	1,091947	1,0243346	3,73715	0,000377	0,0128387	-0,18543
ENSG00000143194	MAEL	1,091832	-0,14465	3,518161	0,000769	0,0216054	-0,72512
ENSG00000133134	BEX2	1,08752	2,5914098	4,233092	6,89E-05	0,0035788	1,244196
ENSG00000124140	SLC12A5	1,086831	-0,536531	4,517607	2,48E-05	0,0015836	2,458433
ENSG00000081052	COL4A4	1,085081	3,2051119	4,704144	1,25E-05	0,0009055	2,798045
ENSG00000184226	PCDH9	1,078831	2,6762872	3,554561	0,000684	0,0197174	-0,92863
ENSG00000273796	NA	1,07787	-0,355147	3,993796	0,000159	0,0068893	0,736369
ENSG00000250986	LINC02600	1,076749	-0,719262	3,285302	0,001594	0,0364162	-1,33493
ENSG00000154258	ABCA9	1,075512	3,4337957	3,903372	0,000216	0,0087262	0,043531
ENSG00000125730	C3	1,073116	9,0793181	3,817159	0,000289	0,0104616	-0,37017
ENSG00000269113	TRABD2B	1,071369	2,7336599	4,136479	9,68E-05	0,0046388	0,904402
ENSG00000146147	MLIP	1,067335	-0,904982	3,234335	0,001863	0,0406465	-1,45879
ENSG00000231672	DIRC3	1,063085	0,3321245	3,21936	0,00195	0,0419141	-1,62011
ENSG00000282164	PEG13	1,059271	-0,762162	4,768692	9,80E-06	0,0007448	3,322072
ENSG00000108924	HLF	1,046203	2,2743008	3,929613	0,000198	0,0081841	0,286402
ENSG00000063127	SLC6A16	1,042938	0,5362319	4,671871	1,40E-05	0,0010007	2,933613
ENSG00000262061	LOC100506388	1,037435	0,9609484	3,744781	0,000367	0,0125658	-0,15544
ENSG00000092096	SLC22A17	1,034366	2,3758218	5,081376	2,99E-06	0,0002979	4,261931
ENSG00000103723	AP3B2	1,028525	0,7363482	3,462216	0,000918	0,0244787	-0,97735
ENSG00000205795	CYS1	1,028416	3,8338645	4,166371	8,72E-05	0,0043155	0,842572
ENSG00000108405	P2RX1	1,027299	2,6065261	3,817078	0,000289	0,0104616	-0,11169
ENSG00000138769	CDKL2	1,024167	-0,21587	3,613972	0,000564	0,017297	-0,43726
ENSG00000223764	LINC02593	1,023297	1,8334759	4,4826	2,82E-05	0,0017434	2,173902
ENSG00000132554	RGS22	1,022656	0,0810003	3,774545	0,000333	0,0116713	0,021114
ENSG00000153253	SCN3A	1,020319	0,6040466	3,283169	0,001605	0,0366089	-1,47363
ENSG00000123999	INHA	1,01845	0,3247752	3,296852	0,001539	0,0354298	-1,4043
ENSG00000016082	ISL1	1,017621	2,5945412	3,578427	0,000633	0,0186115	-0,84584
ENSG00000215252	GOLGA8B	1,017444	5,3072187	3,635445	0,000526	0,0163809	-1,04641
ENSG00000076864	RAP1GAP	1,01246	5,7479441	4,031591	0,000139	0,0062324	0,194091
ENSG00000156414	TDRD9	1,009804	0,5379592	3,78032	0,000327	0,0115163	-0,00344
ENSG00000114115	RBP1	1,009241	5,2433462	3,894096	0,000223	0,0089446	-0,22678
ENSG00000270093	NA	1,005681	-0,605546	3,629125	0,000537	0,0166075	-0,36138
ENSG00000166035	LIPC	1,003823	0,3436342	4,298837	5,46E-05	0,0029265	1,676244
ENSG00000112530	PACRG	1,001799	0,2072737	4,146199	9,36E-05	0,0045967	1,184626

A.

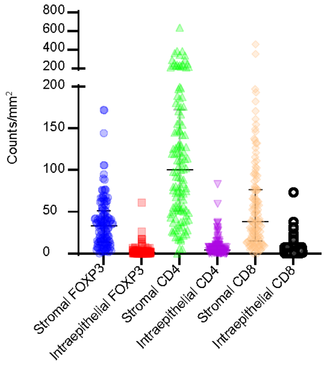

Figure S1. Distribution of immune cells within the different compartments of the tumor microenvironment

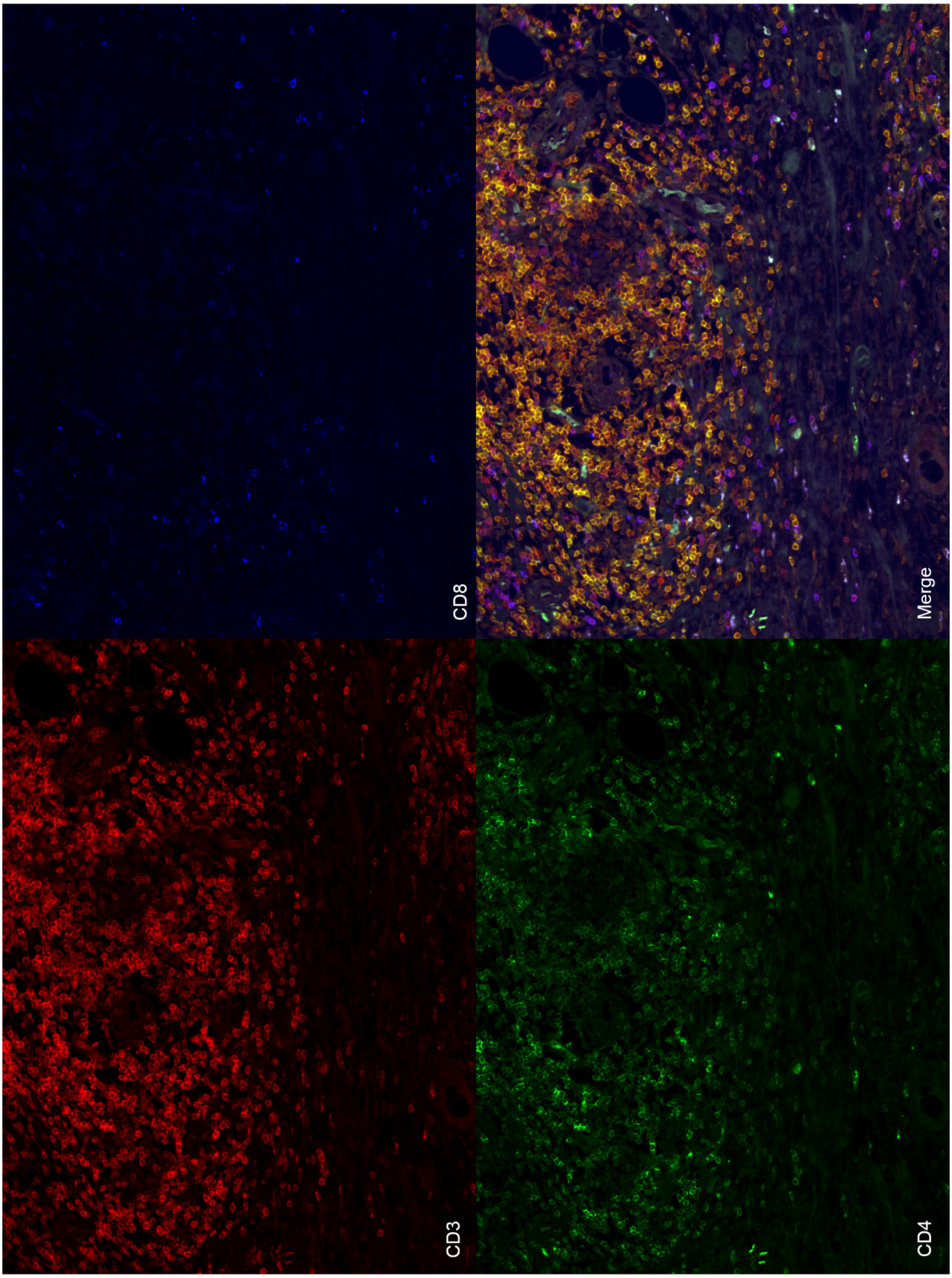

Figure S2. Validation that $CD3^{+}CD8^{-}$ T cells corresponded to $CD4^{+}$ T cells.

a.

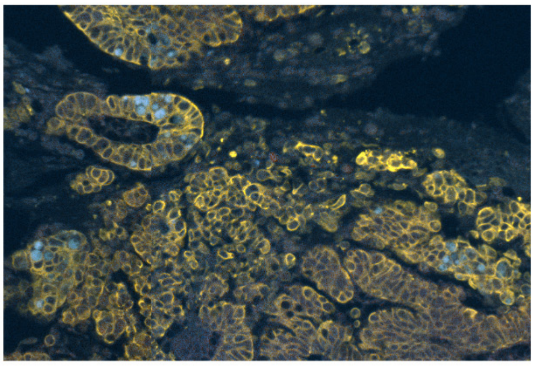

b.

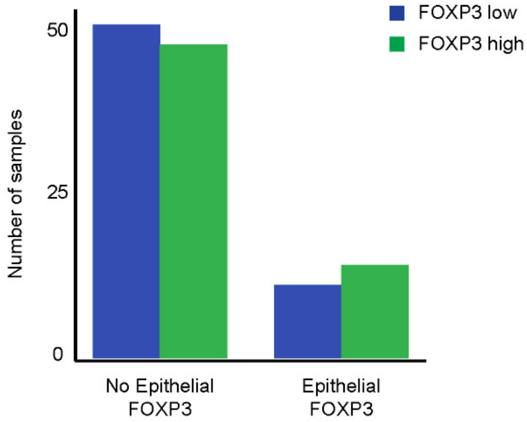

Figure S3. FOXP3 expression on PDAC cancer cells. Representative image displaying FOXP3 in PDAC cancer cells (a). Number of samples expressing FOXP3 in PDAC cancer cells within the FOXP3 high and low infiltrated groups (b).

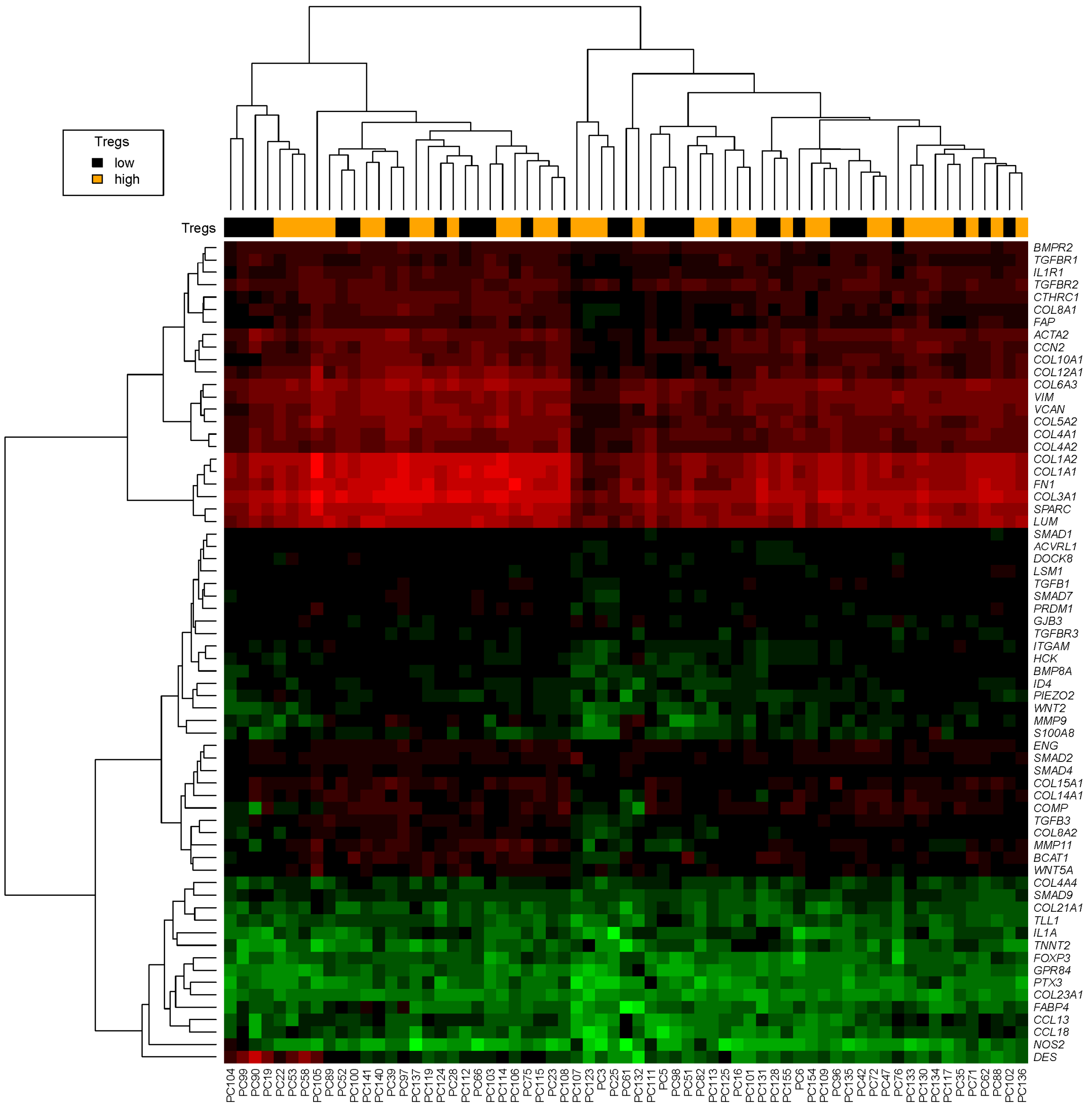

Figure S4. Gene expression profiling investigating differential expression of immune-related genes between Treg-high and Treg-low tumors.

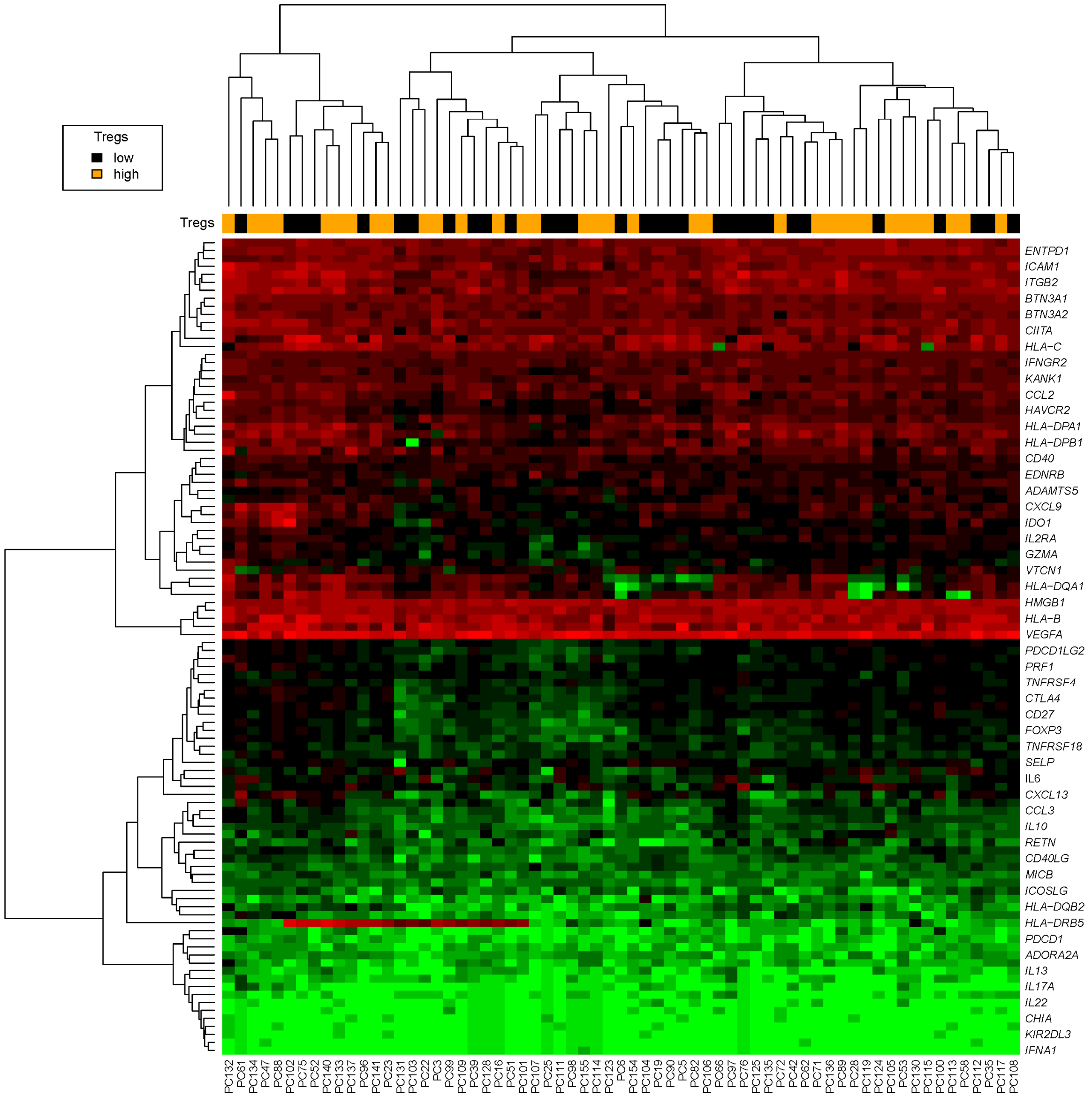

Figure S5. Gene expression profiling investigating differential expression of fibroblast-associated genes and TGF-β signaling targets between Treg-high and Treg-low tumors.

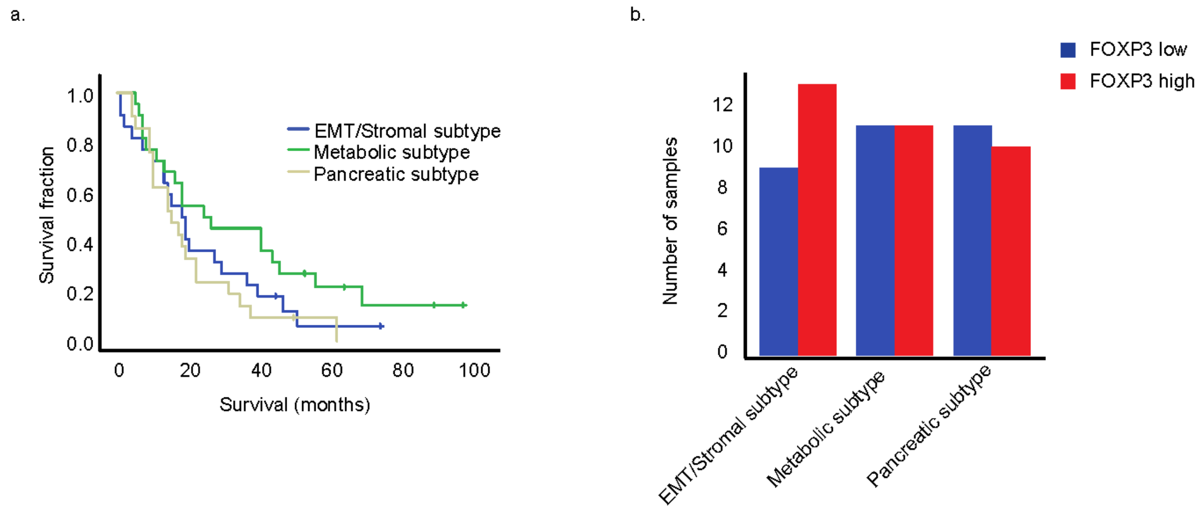

Figure S6. Unsupervised analysis of gene expression profiles identified 3 PDAC subtypes; EMT/stromal subtype (blue); Metabolic subtype (green); Pancreatic subtype (yellow/brown). Kaplan–Meier analysis of patient survival stratified by subtype (a). Number of samples per gene expression profile within the FOXP3 high and low infiltrated groups (b).

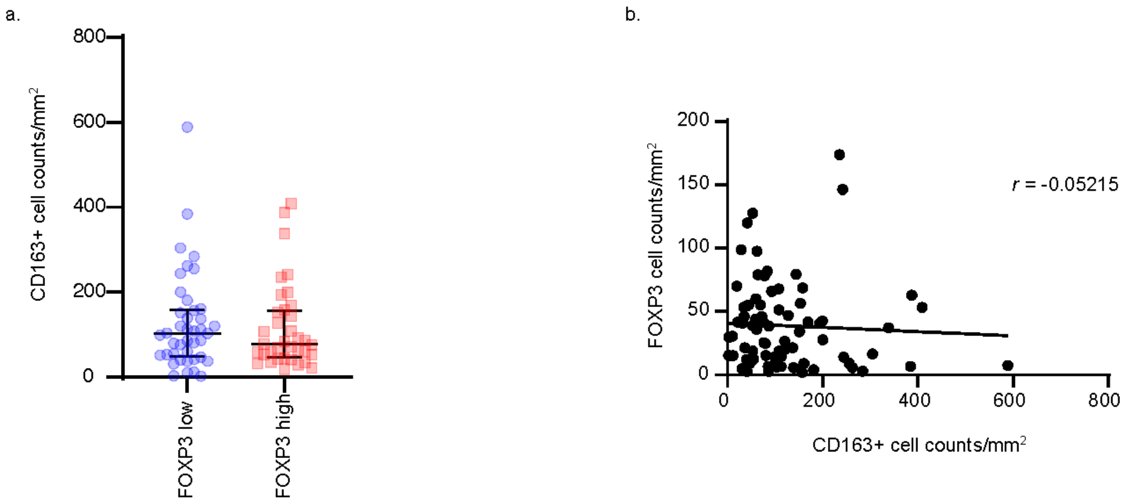

Figure S7. Low Treg infiltration does not result in a compensatory increase of myeloid-driven immune-suppression. Frequencies of analyzed CD163$^+$ cell counts in Treg-high and -low infiltrate groups (a). Correlation analyses between CD163$^+$ counts and CD3$^+$CD8$^-$FOXP3$^+$ counts (b).
